# Role of poly(ADP-ribose) polymerase-1 in regulating human islet cell differentiation

**DOI:** 10.1038/s41598-022-25405-w

**Published:** 2022-12-13

**Authors:** Nidheesh Dadheech, Abhay Srivastava, Rashmi G. Shah, Girish M. Shah, Sarita Gupta

**Affiliations:** 1 Department of Surgery, Alberta Diabetes Institute, University of Alberta, Edmonton, AB T6G 2E1 Canada; 2 Department of Biochemistry, Faculty of Science, The Maharaja Sayajirao University of Baroda, Vadodara, 390021 Gujarat India; 3 Regenerative Medicine Program, Department of Physiology and Pathophysiology, Rady Faculty of Health Sciences, University of Manitoba, Winnipeg, MB R3E 0W2 Canada; 4 CHU de Quebec Laval University Hospital Research Centres Quebec City, Quebec, G1V 4G2 Canada

**Keywords:** Developmental biology, Differentiation, Stem cells, Adult stem cells, Regeneration, Stem-cell differentiation, Endocrinology, Endocrine system and metabolic diseases, Regenerative medicine, Stem-cell biotechnology, Tissue engineering

## Abstract

Poly(ADP-ribose) polymerase-1 (PARP1), a fundamental DNA repair enzyme, is known to regulate β cell death, replication, and insulin secretion. PARP1 knockout (KO) mice are resistant to diabetes, while PARP1 overactivation contributes to β cell death. Additionally, PARP1 inhibition (PARPi) improves diabetes complications in patients with type-2 diabetes. Despite these beneficial effects, the use of PARP1 modulating agents in diabetes treatment is largely neglected, primarily due to the poorly studied mechanistic action of PARP1 catalytic function in human β cell development. In the present study, we evaluated PARP1 regulatory action in human β cell differentiation using the human pancreatic progenitor cell line, PANC-1. We surveyed islet census and histology from PARP1 wild-type versus KO mice pancreas in a head-to-head comparison with PARP1 regulatory action for in-vitro β cell differentiation following either PARP1 depletion or its pharmacological inhibition in PANC-1-differentiated islet cells. shRNA mediated PARP1 depleted (SiP) and shRNA control (U6) PANC-1 cells were differentiated into islet-like clusters using established protocols. We observed complete abrogation of new β cell formation with absolute PARP1 depletion while its inhibition using the potent inhibitor, PJ34, promoted the endocrine β cell differentiation and maturation. Immunohistochemistry and immunoblotting for key endocrine differentiation players along with β cell maturation markers highlighted the potential regulatory action of PARP1 and augmented β cell differentiation due to direct interaction of unmodified PARP1 protein elicited p38 MAPK phosphorylation and Neurogenin-3 (Ngn3) re-activation. In summary, our study suggests that PARP1 is required for the proper development and differentiation of human islets. Selective inhibition with PARPi can be an advantage in pushing more insulin-producing cells under pathological conditions and delivers a potential for pilot clinical testing for β cell replacement cell therapies for diabetes.

## Introduction

Oxidative stress is a primary cause of β cell degeneration and progressive loss of insulin-producing β cells that is a critical pathogenic hallmark of type-1 and -2 diabetes^1^. Poly(ADP-ribose) polymerase-1 (PARP1) protein is an abundant nuclear enzyme in higher eukaryotes implicated in various DNA damage responses ranging from DNA damage repair and cell survival to inflammation and cell death^2–5^. PARP1 is catalytically activated in response to DNA damage to consume NAD and form polymers of ADP-ribose (PAR) that post-translationally modify or PARylate key target proteins and transiently alter their functions until PAR chains are removed from the proteins^6,7^. These reactions, i.e., PARP-activation and its consequences, such as PARylation of proteins and NAD-depletion, have been studied in conjunction with two of the key DNA damage responses, namely DNA repair and cell death^8–10^. Moreover, PARPs have other housekeeping roles in transcriptional activation, repression, or chromatin remodelling during cell differentiation^6,11^. The embryonic stem cells (ESCs) from PARP1^−/−^ mice exhibit altered expression of about 10% of genes compared to only 3% of genes in the liver compared to these cells from normal mice^12^. PARP1 was shown to promote stem cell differentiation by antagonizing the DNA binding transcription factor Sox2 to stimulate expression of the gene encoding fibroblast growth factor 4 (FGF4) that promotes differentiation^13^.

The role of PARPs in diabetes has been explored for the last 25 years^11^ initially using the PARP-inhibitors^14–16^ and more recently using PARP1^−/−^ mice or cells^17–20^. The mechanistic role of PARP1 in diabetes, pancreatic regeneration^21^, and β cell replication in mice^6,22^. Studies in a simpler model of only β-cell lines revealed that PARP-inhibitor could promote transcription of Reg-1^6^ and Maf-A^9^ genes, which are implicated in β-cell proliferation or insulin gene expression, respectively. Two such studies established the role of PARPs in the destruction of pancreatic β-cells in streptozotocin or alloxan models of diabetes and present evidence for direct or indirect oxidative DNA damage which activates PARP1^14,23^. In mice, when PARP function is inhibited^14^ or PARP-gene is knocked out^17,19,20^ pancreas fails to develop diabetes. Additionally, the role of PARP in hyperglycemia-induced diabetic complications^24,25^, such as diabetic changes in endothelial functions^25^, neuropathy^26^ and retinopathy^27^ is well documented. In contrast, the role of PARP in the proliferation of β-cells has also been comparatively less examined^6^. In RINm5F insulinoma-derived β cells, it has been shown that only in its native (unmodified) state PARP can bind in vitro to the promoter region of proliferation-associated gene Reg-1 and increase its expression^6,21^. Finally, the least examined is the role of PARP in insulin biosynthesis. INS-1 β-cells treated with weak PARP inhibitors (*e.g.* nicotinamide) could show an increase in insulin biosynthesis by increasing the expression of gene Maf-A^9^.

The above-described studies highlight the role of PARP in developing diabetes, insulin secretion and biogenesis using isolated pancreatic β cells. However, islet differentiation and cell replacement from stem/progenitor cells also play a crucial role in the development and control of diabetes. Therefore, we examined the potential role of PARP1 in these processes. Here using the model of human islet cell differentiation in vitro from stem/precursor cells, we show that PARP1 depletion by RNAi or pharmacological inhibition (PJ34) regulates the process of differentiation of mature islet cells from precursor cells.

## Results

### PARP-1 null mice show distinct pancreatic and islet architectural differences

**Figure 1:**
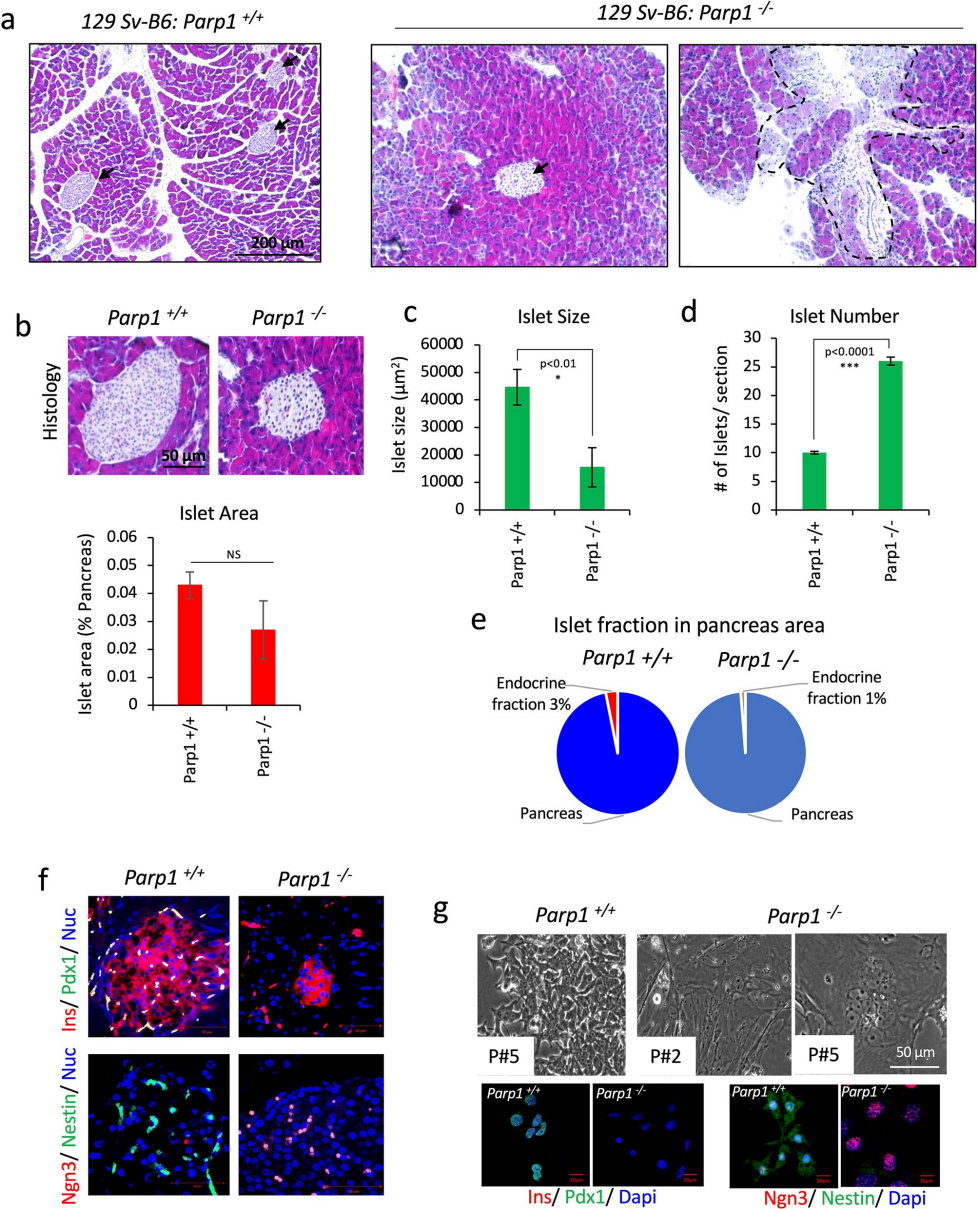
PARP1^+/+^ and PARP1^−/−^ mice pancreas comparative histology, islet quantification, and immune characterization. (**a**) Represents H&E histology of mice pancreas from 129SV-B6 stain PARP1 wild type and PARP1 KO mice. Arrow represents islets, and outline shows a region of ductal fibrosis. (**b**) Depicts pancreatic islet histology between PARP1 wildtype and KO mice and quantification of mean islet area represented as % of the total pancreas (n = 4). (**c**, **d**) Show quantification for mean islet size and number distribution in the pancreas of both genotypes. To assess this, harvested pancreas from 4 animals in each genotype were assessed. Tissue slides from each animal were stained; 3 slides per section from each of the 4 animals were imaged and quantified. Data represented as mean ± sem, p-value show significance at 95% confidence interval using ttest analysis compared to wildtype animals (n = 4). (**e**) Shows the distribution of endocrine fraction within the pancreas between wildtype and PARP-KO mice representing % islet fraction (distribution per unit area) relative to total pancreas area (n = 4). (**f**) Represents immunohistochemistry images for pancreatic progenitor markers- pdx1 (green), nestin (green), ngn-3 (red) and insulin (red). (**g**) Shows brightfield phase contrast microscopic images of PREP cells isolated from wildtype and KO mice pancreas with immunocytochemistry for progenitor markers- pdx1 (green), nestin (green), ngn-3 (red) and insulin (red).

To assess and distinguish between the roles of PARP1 protein and its activity in the stem cell-mediated islet regeneration potential, we first compared pancreatic histology for islet development in PARP1 knockout and wild-type mice produced in the 129SV-B6 strain background. An age-dependent study from Zhang et al.^22^ has reported no effect of PARP-1 deletion on glucose homeostasis and functional regulation of β cells in young and old KO mice despite the whole body PARP1 knockout. Considering non-affected glucose metabolism, we expanded our investigation to delineate the developmental aspects of islet formation and pancreas architecture in PARP1 wild-type and KO mice. Although the islet structure differed morphologically between the two genotypes, the pancreas of PARP1-KO mice showed extensive ductal fibrosis not evident in wild-type mice (Fig. 1a). More remarkable was the difference in islet size and frequency distribution, which were half the size in KO pancreas (Fig. 1b, c), but they were 2× more abundant (Fig. 1d) than islets of wild-type mice. Therefore, the total islet area in KO mice was not significantly different from wild-type mice (Fig. 1b). The smaller size indicates immature islets in KO mice. This was evident when we compared the islet endocrine fraction, which accounted for only 1% of the pancreas in KO mice, unlike 3% mass in wild-type pancreas (Fig. 1e).

Next, we compared the functional and developmental indicators of the islets in these mice. While PARP1^+/+^ islets showed insulin presence (red) that co-labelled with Pdx1 (green) staining within the islets, the PARP1^−/−^ null mice islets, although smaller, showed equally intense insulin staining (red) without accompanying Pdx-1 (green) staining. Furthermore, PARP1^-−/−^ islets show high frequency for master endocrine progenitor marker Neurogenin (Ngn)-3 (red) than PARP1^+/+^ islets that express Nestin- a neuroendocrine filament protein (green) (Fig. 1f). To reconfirm these molecular differences, we isolated and purified pancreatic resident progenitor cells (PREPs) from these mice, as previously detailed by us^28^. The purified PREP cells represented high diversity morphologically and in expansion capacity. While the wild-type PREP cells displayed the expected small non-elongated tightly packed epithelioid morphology with round and tiny nuclei, the PARP1-KO PREP cells showed elongated and fibroblastic morphology and large nuclei structure, suggesting genetic instability (Fig. 1g). In addition, immunocytochemical characterization reconfirms the presence of Pdx-1 (green) staining in PARP1^+/+^—derived PREPs without insulin (red) staining. Similarly, we observed Nestin in PARP1^+/+^—derived PREPs while the same was depleted in PARP1^−/−^ derived PREPs. Staining for Ngn-3 marker was found on both the cells; however, PARP1^−/−^ derived PREP cells showed intense Ngn-3 stained nuclei (Fig. 1g).

Our observations with pancreatic islets from PARP1-KO and wild-type mice, as well as with their PREP progenitor cells, indicate a regulatory role of PARP1 in stem cell differentiation and control of gene expression during the development and function of β cells in the islets. Our results are in agreement with an earlier report that PARP-inhibition results in increased expression of PARP1 in Reg-1 and Maf-A in the partially pancreatectomized (PPx) mice and INS-1 cell line^6,29^.

### PARP-1 depletion impairs islet development and differentiation from the human pancreatic progenitor PANC1 cells

**Figure 2:**
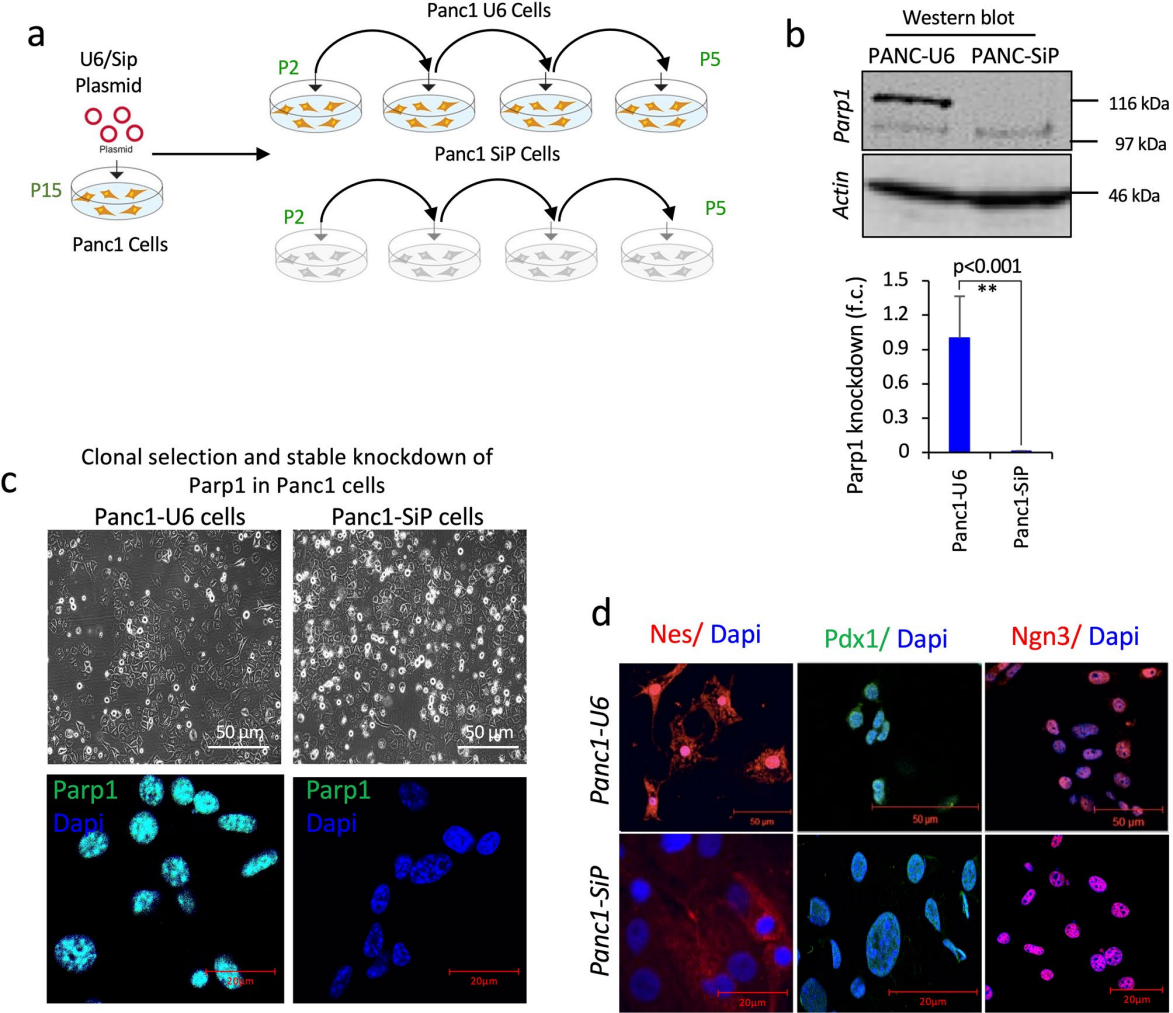
Creation of PARP1^+/+^ (U6) and PARP1^−/−^ (SiP) cells for functional characterization. (**a**) Schematic representation for clone generation, selection, and isolation after PARP1 silencing using SiP and mock U6 shRNA control. Cells for experimentation were confirmed for absolute PARP1 depletion at passage 5. (**b**) Immunoblot confirmation of PARP-1 depletion in SiP and U6 cells. The graph represents quantification for PARP1 depletion in most established clones after 3 independent passages. Data represented as mean ± sem, p-value show significance at 95% confidence interval using ttest analysis compared to U6 cells (n = 3). (**c**) Phase contrast images for PARP1 control and deplete SiP-PANC-1 cells showing morphological changes after KD. Fluorescent staining show images for PARP-1 (green). (**d**) Immunocytochemistry images for pancreatic progenitor- nestin (red), pdx1 (green), and ngn-3 (red) in PANC-1 U6 and SiP cells.

To examine the role of PARP1 in the development of islets from progenitor cells, we used PANC1 cells as the model human pancreatic progenitor cells, which can be subjected to conditions that lead to differentiation and formation of functional islets, as described earlier^30–32^. We knocked down (KD) PARP1 in PANC1 cells by shRNA technique, as described earlier^8^, to create PARP1-depleted PANC1-SiP cell lines and the corresponding PARP1-proficient PANC1-U6 cell lines (Fig. 2a). The immunoblotting for PARP1 in these cells confirmed that PARP1 levels were below the detection limit in SiP cells as compared to its full expression in control U6 cells (Fig. 2b) which was further validated with immunocytochemical staining of PARP1 in these cells (Fig. 2c). Morphologically, there was no noticeable difference in between these two cell lines (Fig. 2c). Still, these cells differed in pancreatic progenitor expression. There was significantly decreased expression of Nestin (red) and Pdx1 (green) in SiP cells compared to U6 cells, while Ngn-3 remained unchanged (Fig. 2d). These observations from KD studies reflect similar changes what we have observed in PARP1^−/−^ mice.

**Figure 3:**
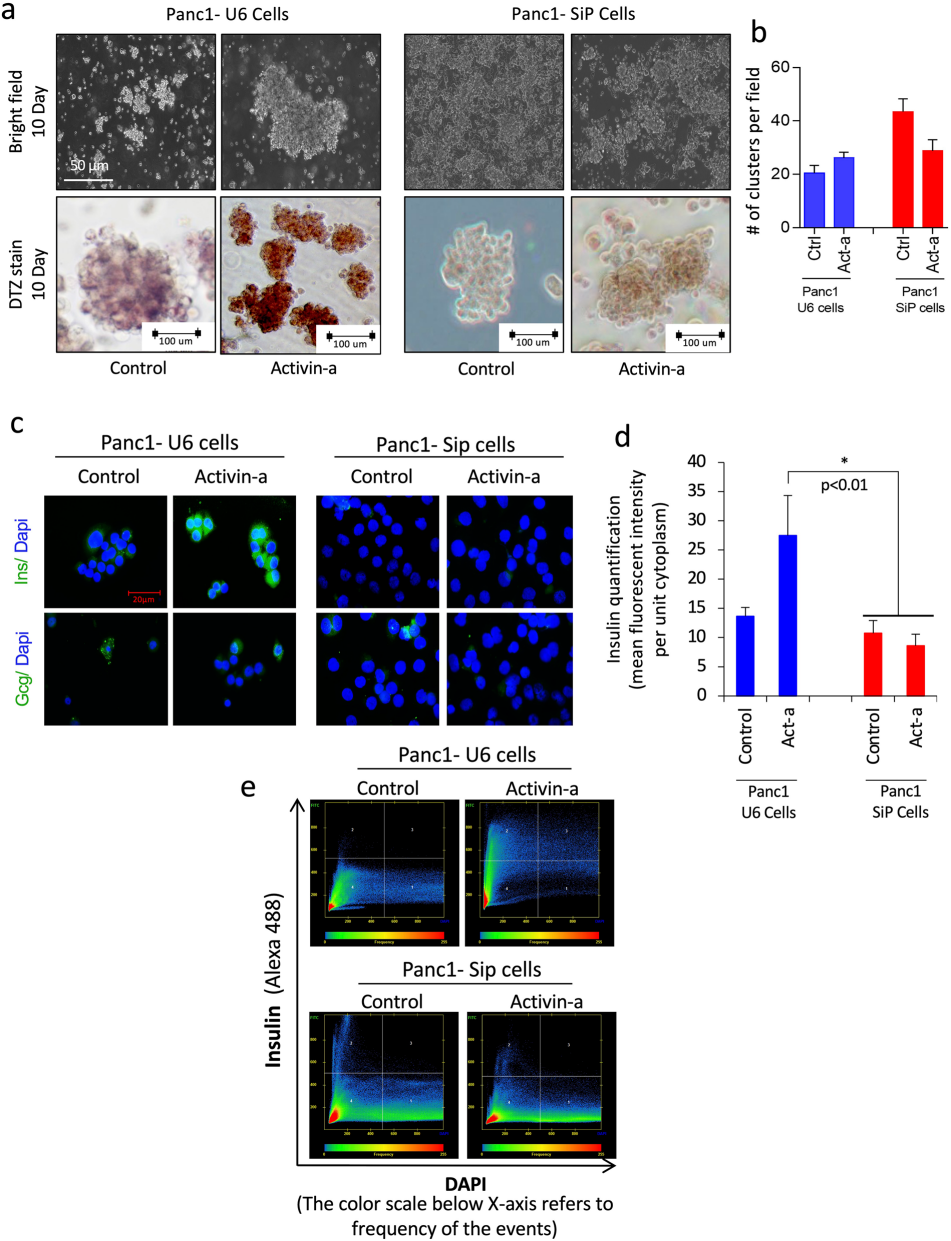
Islet cell differentiation and characterization for insulin production in PARP1 control and deplete PANC-1 cells. (**a**) Phase contrast microscopic images of PANC-1 U6/SiP cells differentiated with control (SFM) and activin-A on day 10th. Color brightfield images show the presence of brick-red insulin staining with dithizone (DTZ) after differentiation. (**b**) Graph show quantification for islet-like clusters generated per field of an image from PANC-1 U6/SiP cells at day 10th differentiation. (**c**) Immunocytochemical characterization of generated clustered from PANC-1 U6/SiP cells for islet cell markers- insulin and glucagon (green). (**d**) Quantification for mean fluorescence intensity for human insulin production in differentiated U6 and SiP cells. Data represented as mean ± sem, p-value show significance at 95% confidence interval using two-way anova analysis compared to U6 cells (n = 3). (**e**) Quantification and modelling for presence of insulin in U6 vs SiP cells using 2-dimensional mean fluorescent intensity dot plot representation extracted from immunostained images with Axio-vision Zen 10 software, Zeiss, Canada, where insulin stays on Y-axis and dapi is marked on x-axis.

These matched pair of progenitor PANC1 cell lines with or without PARP1 were probed for their capacity to differentiate and form islet-like aggregates using established methods and tested growth factor, activin-A, a TGF-β ligand, as has been shown earlier for PANC1 cells^30–33^. We first confirmed with the parental PANC-1 cells treated with activin-A in defined media for 10 days to induce a stage-specific differentiation resulting in islet-like clusters formation. These islet-like clusters displayed an intense islet-specific dithizone (DTZ) staining, and signals for insulin and glucagon were detectable in these clusters by immunostaining (Supplementary Fig.-1a-b), and a semi-quantitative gene expression approach showed insulin and glucagon transcript levels in PANC-1 differentiated cells with activin-A (Supplementary Fig.-1c) confirming the formation of endocrine islets from parental PANC1 cells. Next, we subjected PARP1-SiP and U6 clones to an identical differentiation protocol. Both the cell lines developed formed islet-like aggregate, but the size of SiP-derived clusters was smaller as compared to U6-derived clusters. Moreover, while activin-A treated U6-clusters showed intense staining for DTZ, compared to serum-free media (SFM) control, the SiP-derived clusters failed to stain for DTZ in SFM control and activin-A treatment (Fig. 3a). The reduction in β cell-specific zinc-binding DTZ dye uptake in activin-treated SiP cells indicated a significantly impaired process of islet differentiation in these cells.

Interestingly, quantifying the total cluster yield did not indicate any significant difference between these two cell lines. However, the SiP cell-line-derived clusters were deformed, primarily associated with loosened cells, reduced size and immature architecture compared to PARP1 proficient U6-derived clusters (Fig. 3b). The testing for insulin and glucagon as primary islet markers revealed intense insulin and glucagon staining in activin-A treated U6-clusters compared to weak staining observed in SFM-control U6 clusters (Fig. 3c). In contrast, SiP-clusters failed to stain for both insulin and glucagon in both SFM control, and activin-A treated conditions, (Fig. 3c). Mean fluorescent intensity (MFI) for insulin per unit area of cytoplasm per cell quantification highlight the failed differentiation response with significantly lower MFI in activin-A treated SiP-clusters compared to U6-clusters (Fig. 3d). A pixel-by-pixel densitometric representation of cells using insulin as a surrogate marker confirms the MFI observation. Outright migration of cells density (dot plots) for insulin immunopositivity (Y-axis scale) in activin-A treated U6-clusters. In contrast, negative cell density in SiP-clusters confirms failed differentiation in SiP-clusters (Fig. 3e). These results suggest that the presence of PARP1 protein is essential for normal islet development and differentiation, and its depletion impedes the islet developmental process.

### PARP-1 depletion precludes activin-A induced islet differentiation pathway

**Figure 4:**
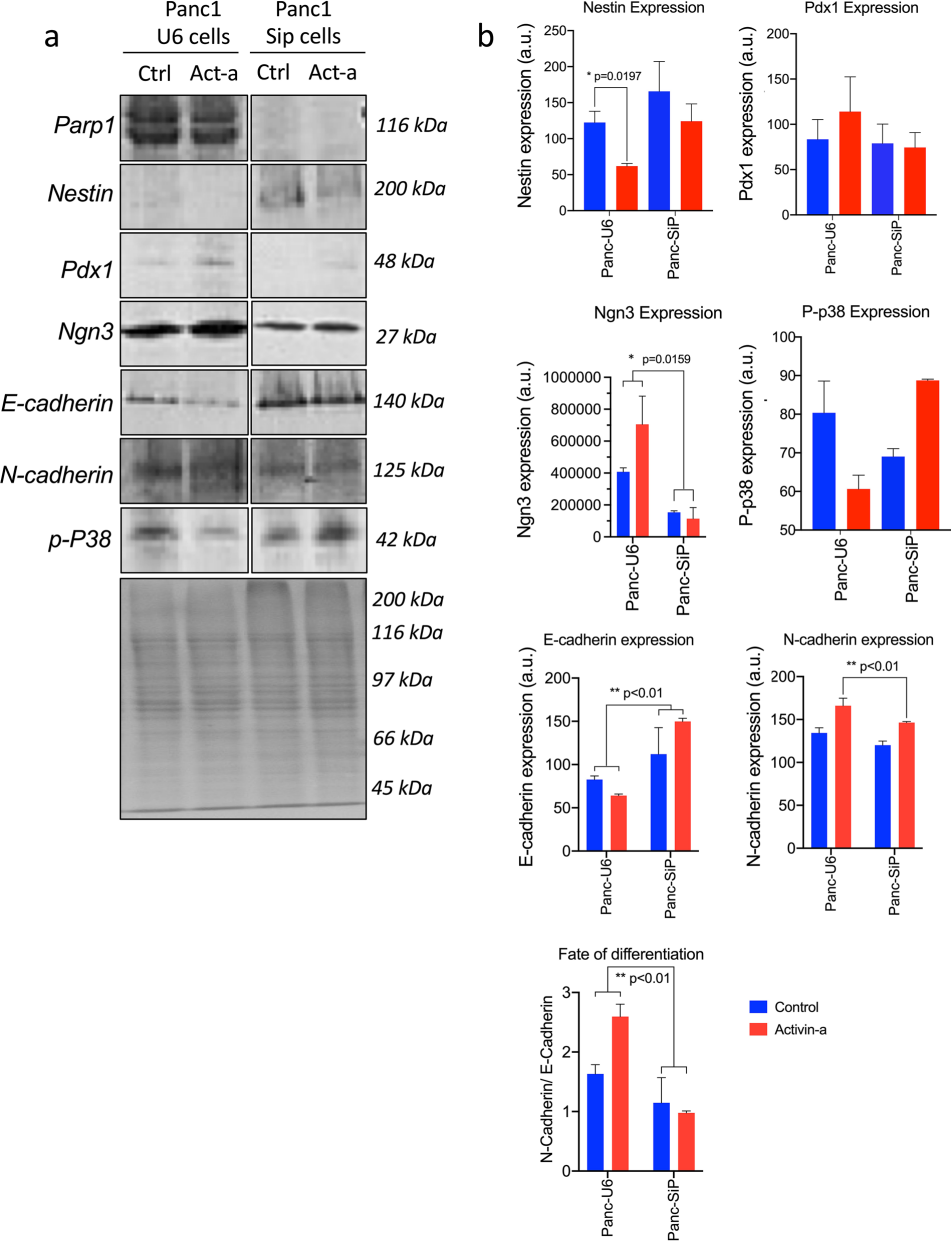
Molecular characterization and quantification of proteomic signals for efficient islet cell differentiation in PANC-1 U6 vs SiP cells. (**a**) Immunoblotting profile of PARP1 expression along with key pancreatic progenitor markers in SFM and activin-A induced differentiation in PANC-1 cells. (**b**) Quantification for endocrine reprograming transcription factors and ratio of progenitor (E-cadherin) states vs endocrine β cell (N-Cadherin) states. Data represented as mean ± sem, p-value show significance at 95% confidence interval using two-way anova analysis compared to U6 cells (n = 3).

Next, we explored the mechanism of action of PARP1 during β cell differentiation process. Earlier, we showed that activin-A during islet cell differentiation is attributed to early and accelerated stimulation for Pdx1-Ngn3 activation that allows for directed initiation of the p38 MAPK-TKK pathway and a downstream signal transduction cascade that facilitates endocrine cell maturation^30^. Here, we evaluated the effect of PARP1 on the Ngn-3 activated p-38 phosphorylation pathway in both U6- and SiP-derived clusters differentiated under SFM control and activin-A treatment. As expected, in U6 cells, activin-A initiated Ngn-3 upregulation translated to phospho-p-38 downregulation (Fig. 4a). Since Ngn-3 activated p38 phosphorylation event is short-lived, on day 10th, differentiated islet cells undergo maturation phase and therefore represents lower p-38 phosphorylation levels. Moreover, this Ngn3 upregulation changes protein expression by impeding Nestin progenitor marker expression while promoting Pdx1 levels. In parallel, this regulates the cytoplasmic cytoskeleton remodelling by blunting E-cadherin while effectively augmenting N-cadherin expression (Fig. 4a, b). In contrast, SiP cells exhibited high Nestin expression without Pdx-1 and Ngn3 induction despite activin-a treatment. The cells remained quiescent in epithelioid E-cadherin expression progenitor state while minimal N-cadherin expression was detected (Fig. 4a). Quantification for these protein markers clearly highlighted the significantly high E-cadherin expression retained in SiP-differentiated cells compared to U6-cells, while Ngn3 and N-cadherin expression was largely abrogated after 10-day differentiation (Fig. 4b). Thus, our mechanistic evidence endorses PARP1 involvement and direct regulatory action in regulating master endocrine transcription factors, which are indispensable for islet formation.

### PARP-1 action for islet differentiation is independent of its activation

**Figure 5:**
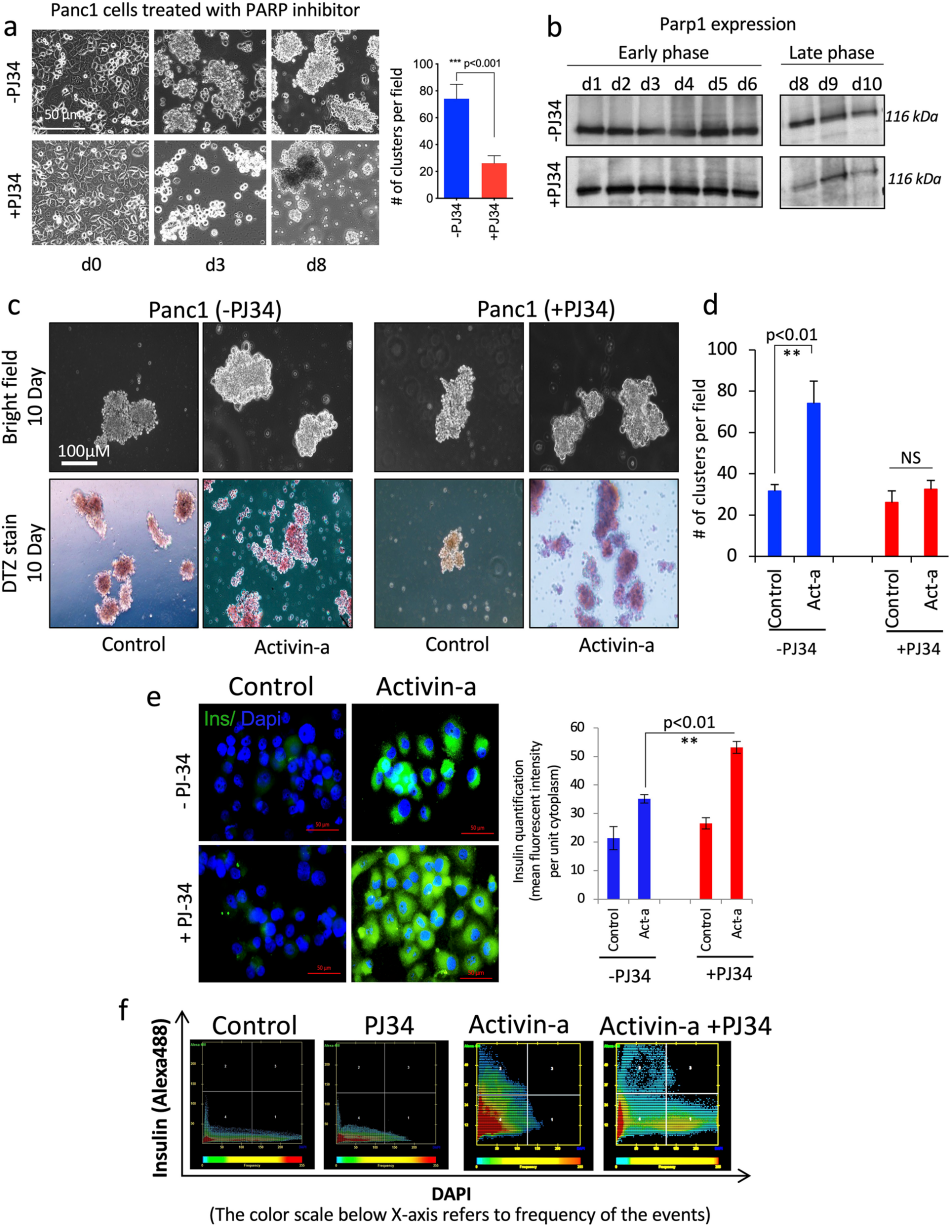
Islet cell differentiation and characterization in PANC-1 cells using PJ34 mediated PARP1 inhibition. (**a**) Phase contrast microscopic images of PANC-1 cells treated with PARP1 inhibitor PJ34 at day 0, 3rd and 10th. Graph show quantification of cell aggregation or clusters generated per field of image from PANC-1 cells with PJ34 treatment at 10 days of treatment. (**b**) Immunoblot profiling for PARP1 protein following PJ34 inhibition at short and long-term exposure. (**c**) Phase contrast microscopic images of PANC-1 cells differentiated with control (SFM), activin-A, and activin-A with PJ34 in combination at day 10^th^ of differentiation process. Color brightfield images show presence of brick-red insulin staining after differentiation using dithizone (DTZ) stain. (**d**) Quantification for islet-like clusters generated per field of image from PANC-1 cells in control, activin-A and activin-A with PJ34 treatment after 10 days of differentiation. (**e**) Immunocytochemical characterization of generated clustered from PANC-1 cells followed activin-a and PJ34 treatment for insulin (green) to confirm β cell differentiation. Graph show quantification for mean fluorescence intensity for human insulin production in differentiated cells. Data represented as mean ± sem, p-value show significance at 95% confidence interval using two-way anova analysis compared to activin-A differentiated cells (n = 3). (**f**) Quantification and modelling for presence of insulin in activin-a differentiated cells with and without PJ34 using 2-dimensional mean fluorescent intensity dot plot representation extracted from immunostained images with Axio-vision Zen 10 software, Zeiss, Canada, where insulin stays on Y-axis and dapi is marked on x-axis.

To further assess the involvement of PARP1 protein per se or the dependency of its catalytic action in islet cell differentiation, we aim to test PARP1 facilitated islet differentiation action in the presence and absence of strong PARP pharmacological inhibitor- PJ34. The aim is to complement the observed regulatory effects of PARP1 depletion via knockdown in PANC-1 cells prior to islet differentiation using the DNA vector-based RNAi approach. We tested islet cell differentiation using PARP1^+/+^ PANC-1 cells adopting an identical 10-day differentiation protocol using SFM-control and activin-A with and without in combination with PARP-inhibitor, PJ34. Prior to differentiation, we first tested the effect of PARP1 inhibition on the clustering efficiency of PANC-1 cells without differentiating agent activin-A. The central idea behind this was to rule out the possibility of misinterpreting the impact of PARP1 inhibition on differentiation capacity and islet yield. With a 10-day treatment, we found aggregation capacity in PANC-1 cells both with and without PJ34. However, the addition of PJ34 did enormously impact the size and generation of cell clusters, similarly as previously observed with PARP1-depleted SiP cells (Fig. 5a). This was corroborated with cluster yield quantification that displayed a significantly lower yield of clusters (~three-fold less) with the PJ34 treatment. Although similar to PARP1 delete cells, this steep decline was surprising with PJ34 inhibition as PJ34 exerts complete inhibition of the enzyme while unmodified PARP1 protein remains available during the course of differentiation. To refute the possibility of proteolytic cleavage and clearance of PARP1, mimicking knockdown condition, we tested and immunoblotted protein lysates from the PANC-1 treated with and without PJ34 in a 1 to 10-day time-course manner. Surprisingly, we did not observe any significant changes in PARP1 protein levels and effectively ruled out the possibility of proteolytic cleavage or clearance of PARP1 during the 10-day treatment (Fig. 5b). Although inactivated PARP1 protein was highly detected in PJ34 treated cells, the resulting decline in cell cluster yield, however, remained unexplainable.

We then examined the effect of PJ34 on islet cell differentiation into islet-like clusters with and without PJ34 and in combination with activin-A using DTZ staining as a tool to detect the presence of insulin. As expected, cells treated with activin-A but without PJ34 and SFM control showed positive DTZ staining in generated clusters, while activin-A treated cells in combination with PJ34 surprisingly were more intensely stained for DTZ staining, suggesting high insulin content and mature islet cell differentiation (Fig. 5c). The qualification of islet clusters with PJ34 in combination with activin-A displayed a significant reduction in the yield compared to activin-A alone cells. However, this should not be considered as the negative impact of PARP1 inhibition on islet differentiation and maturation as this was primarily associated with PANC-1 cells clustering response to PJ34 prior to differentiation (Fig. 5d).

In parallel, we assessed the differentiated cells with and without PJ34 using islet cell marker immunocytochemistry. As anticipated, activin-A but without PJ34 treated cells profoundly presented high insulin immunopositivity at the 10th day of differentiation compared to control SFM cells. Most interestingly, the magnitude of this insulin immunopositivity was profoundly detected after the activin-A treatment was combined with PJ34 (Fig. 5e). MFI for insulin per unit area of cytoplasm per cell quantification clearly denoted PJ34 promoted islet differentiation response with significantly high MFI in activin-A treatment combined with PJ34 inhibition, compared to activin-A treatment alone (Fig. 5e). A pixel-by-pixel densitometric representation of quantified cells using insulin as a surrogate marker corroborated the MFI observation. Improved migration of cells density (dot plots) for insulin immunopositivity (Y-axis scale) in activin-A with PJ34 compared to activin-A re-establish the clear proof of evidence for improved and accelerated maturation of PANC-1-differentiated islet cells with PARP-1 inhibition (Fig. 5f). These results collectively support the notion of PARP1 exemplary role in controlling the normal islet development and differentiation during stem cell-derived islet regeneration.

### PARP-1 inhibition enhances and accelerates the islet differentiation pathway by augmenting the MAPK pathway

**Figure 6:**
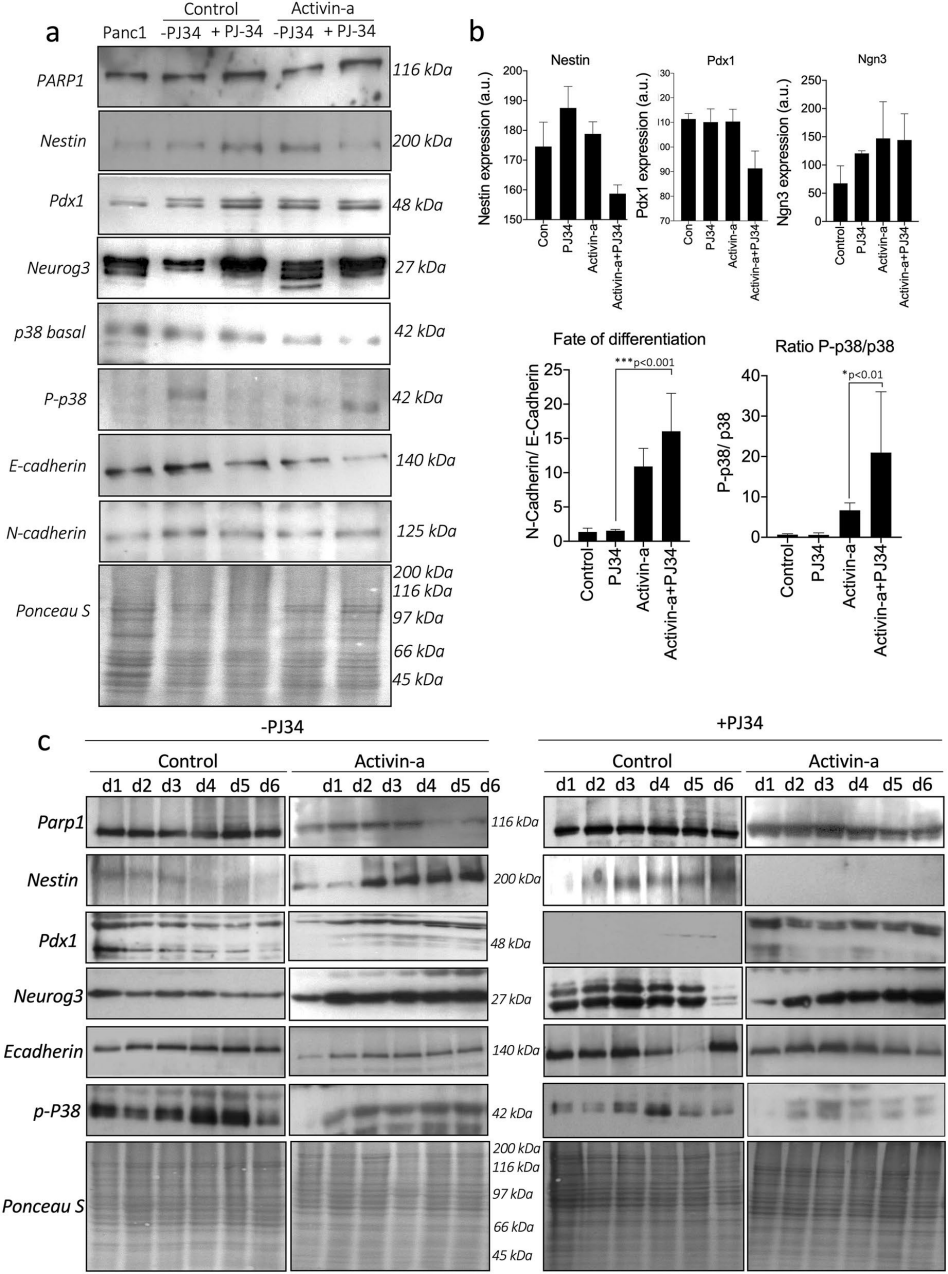
Molecular characterization and quantification of proteomic signals for efficient islet cell differentiation in activin-A treated with and without PJ34 treatment in PANC-1 cells. (**a**) Immunoblotting profile at day 10th of differentiation of PARP-1 expression along with key pancreatic progenitor markers and activin-A induced endocrine pathway proteins that governs pancreatic islet formation from PANC-1 cells. (**b**) Quantification for endocrine reprograming transcription factor, ratio of progenitor (E-cadherin) state vs endocrine β cell (N-Cadherin) state, and MAPK signal (p-p38/p-38) stimulation. (**c**) Short-term (d1-6) protein profiling of differentiation signals initiated within activin-A treated cells with and without PJ34. Data represented as mean ± sem, p-value show significance at 95% confidence interval using two-way anova analysis compared to activin-A cells (n = 3).

To understand the mechanism of action and forecast the profligate impact of PARPi in accelerating the islet cell differentiation pathway, we conducted immunoblot profiling of key endocrine differentiation transcription factors. Protein profiling of differentiated cells on the last day of differentiation (10th day) demonstrated improved cell differentiation and endocrine maturation in cells subjected to combined exposure to activin-A with PJ34 inhibition. As observed earlier, no remarkable change was observed in PARP1 levels after 10-day differentiation in any group. Strikingly, pronounced Ngn3 upregulation and activation with PARPi deliver higher p-38 MAPK phosphorylation and elevated Pdx-1 levels in activin-A-PJ34 treated cells, greater than activin-A alone on 10th day (Fig. 6a). The evidence suggests enhanced flux for endocrine differentiation and maturation with PARPi. Additionally, significant retardation in the E-cadherin signal was also recorded leading to upregulated N-cadherin expression, suggesting improved cytoskeleton remodelling from progenitor to endocrine mature islet cells with activin-A and PJ34 treatment (Fig. 6a). Relative densitometric quantification of N-cadherin to E cadherin suggested an improved and accelerated fate of differentiation in PANC-1 cells treated with activin-A and PJ34 communally. Further, significantly elevated (three-fold) Ngn3 expression rendering elevated p38-MAPK phosphorylation levels (> 20 fold) intensely supported the improved maturation and endocrine differentiation (Fig. 6b). To validate and understand the mode of action for PARPi with PJ34, we further surveyed the MAPK signaling action in a time-course dependent manner during the early phase of endocrine differentiation (i.e. days 1–6). Our results indicated early activation (d3-6) of progenitor marker- Nestin with activin-A treatment, while Nestin was highly downregulated under PJ34 inhibition leading to Ngn3 upregulation and p-38 phosphorylation. The early induction of endocrine signaling with the addition of PJ34-mediated PARP inhibition complemented the action of activin-A for islet cell maturation (Fig. 6c). In fact, the combination of PJ34 with activin-A controls the transcription factors (Nestin and Ngn3) activation and p-38 phosphorylation within 3 h of treatment in PANC-1 cells until 9 h than controls (supplementary Fig. 2).

## Discussion

Oxidative stress is a hallmark indicator of β cell damage in diabetic conditions, and Poly(ADP)Ribose Polymerase-1 (PARP1) protein is a fundamental factor for the repair of DNA in such stress conditions^11^. The role of PARPs in diabetes has been explored for the last thirty years, with early studies employing PARP-inhibitors in isolated β cell function^15,34^, while later reports investigated the action using PARP1^−/−^ mice or derived cells^17–20^. The prime emphasis was, however, overlaid on PARP1 for exploring the role in DNA repair during β cell death, survival, physiology, and insulin secretion^6,22,29^. Moreover, most minor attempts were made to understand the possible mode of action of PARP1 in β cell regeneration or stem cell-derived differentiation. Islet neogenesis is one such pathway for new islet cell development and is highly dependent on the spatial–temporal expression of key transcription factors. This has been very recently established that PARP activity and DNA-PARP Protein interaction conversely modulate a key transcriptional factor involved in islet differentiation pathways such as Sox2, Ngn-3, PDX-1, MafA, Reg-1 etc^6,29^. As the concept is pretty new and relatively unexplored, in the present study, we aim to investigate the role of the PARP1 enzyme in human islet differentiation using a pancreatic progenitor cell line model- PANC-1 to mimic the embryonic islet developmental pathway. Our ultimate aim was to delineate the mechanistic action of PARP1 regulation in new islet formation either by its catalytic activity or independently due to the presence of unmodified protein per se. We performed routine islet differentiation using human progenitor cell line, PANC-1 and activin-A as established differentiating agents by employing catalytic inactivation of PARP using potent pharmacological PARP inhibitor- PJ-34 or attaining absolute protein loss using DNA vector-based RNAi technology.

Our intention in abrogating all PARP enzyme activity using PJ-34 was to answer if PARP1 protein presence is indispensable to facilitate islet neogenesis with activin-A. Additionally, using stable knockdown of PARP1 protein with shRNA, we tested the effect of PARP-1 absence on the islet cell differentiation pathway. Additionally, pharmacological PARP-inhibitor- PJ-34 is a potent nonspecific inhibitor that blocks PARP1 and PARP2 while all other isoforms of PARPs are allowed to carry out DNA repair function, thereby allowing cells to have free access to unmodified PARP protein for molecular interaction with targeted transcription factor proteins in modulating endocrine differentiation other than DNA damage induced repair mechanism^8,10,34–36^. In our study, PJ-34 alone does not impact the islet neogenesis pathway. Still, in combination with activin-A, the PARPi effect was highly pronounced to show accelerated islet clusters differentiation and maturation that was translated to hormone (insulin and glucagon) production. This is evident since the SFM control medium is not rich in growth factors and quickly causes terminal mild-to-severe DNA damage due to starvation, leading to PARP over-activation and catalytically activating the auto modification domain. On another side, PARPi with PJ-34 facilitates free but unmodified PARP protein and allows for direct interaction and recruitment for essential endocrine transcription factor machinery for improved islet differentiation. Because of the catalytic inactivation and its unmodified structure, free PARP1 interacts with regulators including Pdx-1, Ngn-3 and p38 MAPK upon phosphorylation. It became interesting to record the mechanism for the complementary effect of PARP-1 action since this remained largely unstudied, despite a promise for PARPi in clinical trials^15^.

In parallel, separately to PJ-34 inhibition that highlighted the role of PARP1 protein in stimulating islet cell formation, we adopted a reverse strategy to completely abrogate PARP1 levels in the differentiated cells using the gene knockdown approach. The idea was to test whether the absence of PARP1 protein is required for islet formation. To investigate this, we used the DNA vector-based RNAi approach, previously established by Shah GM et. al.,^8^, to stably and selectively knock down PARP1 in PANC-1 cells (PANC-SiP cells) compared to control PANC-U6 cells. We observed that PARP-depleted SiP cells lose the capacity to form insulin-producing cells when treated with activin-A. In contrast, PARP1-control U6 cells could still form routine islet-like clusters that were intensely positive for insulin and glucagon hormones, as measured by DTZ staining and immunocytochemistry. Strikingly, PARP1-depleted SiP-differentiated cells displayed reduced cluster size and retarded islet yield despite activin-A exposure. A mechanistic blueprint from key transcription factors captured after immunoblotting Sip and U6-derived cells clearly reflected the involvement of PARP1 protein in endocrine cell differentiation, confirming the indispensable presence of PARP1 for islet cell differentiation and maturation for 10 days differentiation protocol. The study also shows an elevated expression of progenitor markers like Nestin and E-cadherin in PARP1 depleted cells, indicating that they failed to differentiate, unlike PARP-control U6 cells. Interestingly, activin-A treatment upregulated phospho-p38 in SiP cells, but this was insufficient to translate the strong up-regulation of Ngn-3 and insulin expression. The data from PARP1-depletion in SiP cells and PJ34 inhibition collectively suggest that PARP1 protein is crucial and indispensable for normal islet development and differentiation from stem/ progenitor cell-derived islet regeneration. In Contrast to PARP1 KO animals where Ngn3 expression was observed without nestin staining, RNAi-induced PARP1 SiP cells showed basal nestin trigger without stimulating Ngn3 activation. We believe that Nestin and Ngn3 arm for human islet cell differentiation and maturation might progress distinctly than in mice pancreas development, such as in PARP1-KO mice. Although RNAi-mediated PARP1 knockdown could suppress nearly > 95% of protein expression, a minimal residual protein may influence basal signal transduction but is incapable enough to stimulate ngn3 activation and induce islet cell differentiation. Our data from this study concludes that the absence of PARP1 in KO mice defines immature islets with increased but small size suggesting halted progenitors during morphogenesis. This was not observed when mimicked with RNAi-mediated PARP1-depleted human PANC-1-islets.

**Figure 7:**
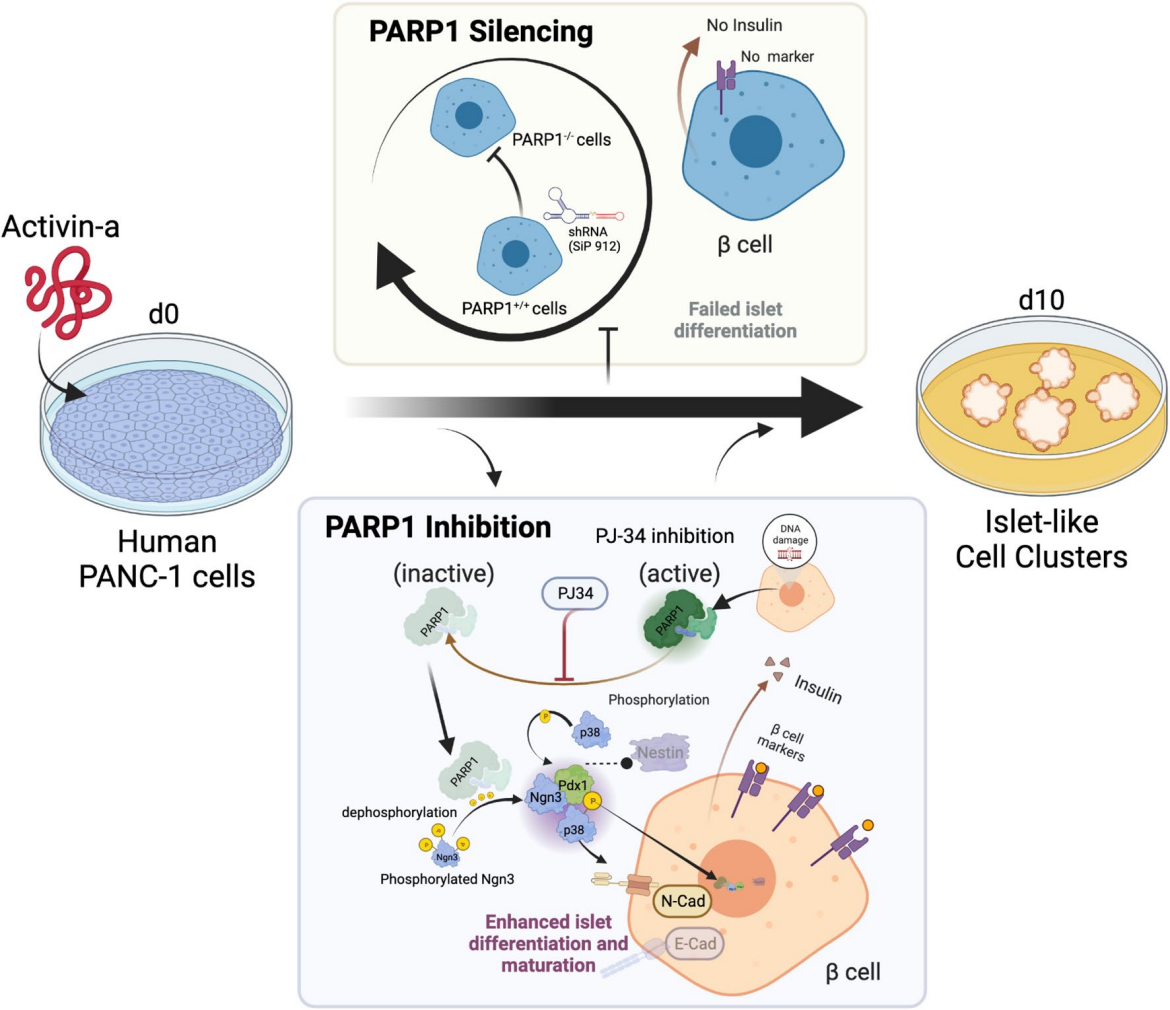
Conceptual illustration describing the mode of action of PARP-1 function in enhancing and activation of islet cell differentiation, maturation and improved β cell production with PJ-34 inhibition while completed abrogation of the process with RNAi mediated PARP1 silencing. The illustration is created using bioRENDER online tool (https://biorender.com/).

Based on these substantial datasets using PARP1 KO mice and PANC-1 line differentiated islets with pharmacological and gene manipulation approach, we hypothesize that the presence of PARP1 protein, but not its catalytic activation, is essential for forming functional islet clusters with activin-A (summarized data shown in Table 1). We also suggest that PARP-activation may negatively influence this event (Fig. 7). Our results reflected the two earlier observations in a simpler model of only β-cell lines that PARP-inhibitor could promote transcription of Reg-1^6^ and MafA^29^ genes, which are implicated in β-cell proliferation or insulin gene expression, respectively. Unlike these studies that involved only mature β cells, our model requires differentiation of precursor cells to islet-like clusters for insulin and glucagon-producing cell generation, thereby mimicking the embryonic pancreatic ontology for islet development artificially. In addition, as PARP is known to have a role in chromatin remodelling and transcriptional activation or repression in different contexts of the genome, we believe that unmodified PARP1 is implicated in transcriptional reprogramming of many genes that govern optimal islet formation from stem/ progenitor cells.

**Table 1:** Table summarizing all markers expression in Mice Vs PREPs Vs PANC-1-Islets.

Markers (High+++ or Low +)	Mice pancreas		PREP cells		PANC-1-Islets	
	PARP-1+/+	PARP-1−/−	PARP-1+/+	PARP-1−/−	PARP-1+/+	PARP-1−/−
Nestin	+++	++	+++	−−−	+++	−−
Pdx1	+++	−−−	++	−−	++	−−
Ngn3	++	−−−	+++	−−	++	+++
PARP-1	+++	‡	+++	†	+++	†
Insulin	++++	−−	+++	−−	+++	−−

+++, very high; ++, high; −−−, very low; −−, low; ‡, knockout; †, knockdown expression.

## Conclusions

Poly(ADP-Ribose) polymerase-1 show necessary regulatory action in controlling islet differentiation and is inevitable for new β cell formation. To further understand the role of PARP1 (catalytic activity or unmodified protein) in controlling the translational processing of essential endocrine regulators-Pdx-1, Ngn-3, NeuroD1, MafA and Reg-1, further molecular studies should be conducted using gain and loss-of-PARP-1 function. Our study suggests that PARP1 is required for the proper development and differentiation of human islets. Selective inhibition with PARPi can be an advantage in pushing more insulin-producing cells under pathological conditions and delivers a potential for pilot clinical testing for β cell replacement cell therapies for diabetes. Future exploration to harness the beneficial effects of PARP1 and PARPi in clinical settings is needed.

## Materials and methods

### Cell culture maintenance and differentiation

Human PANC-1 cells were cultured (Originally procured from ATCC and given as a kind gift from Dr. Girish M Shah’s Lab. Université Laval, Quebec, Canada) and maintained in high glucose DMEM supplemented with 10% Fetal Bovine Serum. PANC-1 cells were allowed for differentiation in the presence of activin-A using a 20 ng/ml dose as previously described^31,32^. After differentiation, islet-like clusters were tested for the presence of insulin with Dithizone (DTZ) staining. The study is performed and reported in accordance with ARRIVE guidelines.

### Animals

Four young (6–8 week) male PARP1 gene-deficient and wild-type SV129 mice^37^ were procured from Jackson laboratories and used for harvesting pancreases for performing a histological examination of islet morphometry and islet distribution census. The animals were not treated in any manner and were kept in 12 h light cycle with regular chaw and water supply. All animal experiments were performed in accordance with animal ethical guidelines and after obtaining institutional committee approval from Université Laval, Quebec, Canada.

### Creation of PREP cells from PARP-1 wild-type and knockout mice

Mice pancreatic endocrine resident progenitor cells or PREP cells were isolated and cultured from PARP1^+/+^ and PARP-1^−/−^ mice for examining the effect of PARP1 KO in maintenance and phenotypic identity of pancreatic progenitor cells. PREP cells were isolated and cultured using the established protocol described in previously published report^28^.

### Creation of Panic-1-PARP-1^−/−^ cells using stable RNA*i* knockdown technology

PARP1 gene was knocked down using specific shRNA against the PARP1 gene. DNA vector-based RNAi approach, established in Dr. Girish M Shah's lab^8^ was used to stably knockdown PARP1 in human PANC-1 cells (SiP) along with empty vector control PARP1-shRNA control (PANC-1-U6 clone), without shRNA. The vector map and shRNA sequence chosen are shown in Fig. 2a.

### Transfection

PANC-1 cells were transfected with plasmid pBS/U6 harbouring PARP shRNA. Transfection was done using lipofectamine 2000 with a DNA to lipofection ratio of 1:2.5. Cells were cultured and selected for positive PARP1^−/−^ clones on hygromycin at the concentration of 200 μg/ml. Clones were purified from isolated colonies using clonal disc selection and further scaled up in 10 cm dishes. An absolute knockdown for PARP1 in the isolated clones was confirmed using Immunoblotting using an anti-PARP-1 antibody. The best clone that displayed more than 90% knockdown was maintained, stocked, and frozen in LN2 till further experimentation use.

### Differentiation into islet-like clusters

Normal PANC-1 cells were differentiated using activin-A (20 ng/ml) as differentiating factors in the eight-day differentiation protocol described by Dadheech et al.^31^. In order to better comprehend the differentiation procedure of islet neogenesis, differentiation of PANC-1, in presence and absence of PJ-34 (Inhibitor of PARP activity) at a concentration of 10 μM. PARP-U6 control and PARP-Sip deplete cells were also differentiated in the same 4-step protocol in the presence of activin-A at 20 ng/ml concentration for 10 days. Islet-like clusters from all groups were purified and counted for yield assessment and stained for DTZ and insulin immunostaining on the 10th day. Islet clusters from each plate were then processed for total protein isolation and immunoblotting of key transcription factors.

### Tissue histology

WE harvested four mice pancreas, fixed and sliced from each genotype. For morphometry and quantification, we used a minimum of 3 slides per animal per genotype. Each slide was stained with hematoxylin and eosin for at least one whole pancreas section and imaged to count for islet structures. All morphometric analyses and quantification were then performed using the ImageJ/Fiji software.

### Immunofluorescence and immunohistology

For understanding the role of PARP1 in islet differentiation, immunohistochemistry for insulin and glucagon and key islet differentiation markers were performed. Clusters obtained post differentiation of PANC-1 cells with and with PJ-34 treatment and after knockdown of PARP1 using Sip 912, and U6 plasmids were stained for insulin and glucagon. Briefly, clusters were collected on the 10th day and allowed to adhere to serum (FBS) coated glass coverslips for 4 h. Once adhered, the clusters were fixed with ice-chilled methanol for 10 min at 4 °C. For mice tissue histology, paraffin fixed and microtome pancreatic tissue sections were first deparaffinized and rehydrated as described previously^31^ followed by blocking and staining for primary antibodies. Both tissue slides and clusters were then permeabilized with 0.25% Triton X 100 and transferred to the blocking solution before primary antibody staining. Primary antibodies, as described in Table 2, were used to stain tissue slides or clusters for 18 h at 4 °C in a humidified chamber. Labelled conjugated secondary antibodies were then used to counterstain primary antibodies, as described in Table 2. Nuclear stain- DAPI was used at 300 nM concentration, and samples were finally mounted with vecta-shield mounting media. Fluorescent images were captured on a Zeiss Axio-Vision Fluorescent microscope (Carl Zeiss, Germany) using Axiovision Zen 10 software.

**Table 2:** List of antibodies used in the study.

Antibodies	Cat#	Isotype IgG	Poly/Mono	Application	Dilution
PARP-1-F2	Santacruz Sc-8007	Mouse	Mono	WB/IF	1:500; 1:800
Nestin	Sigma N5413	Rabbit	Poly	WB/IF	1:1000; 1:500
Pdx1-	BD 554655	Mouse	Mono	WB/IF	1:1000; 1:200
Neurogenin-3	Sigma SAB1306585	Rabbit	Poly	WB/IF	1:1000; 1:100
Insulin	CST 4590	Rabbit	Poly	IF	1:100
Glucagon	Sigma 2654	Mouse	Mono	IF	1:100
C-Peptide	CST 4393	Rabbit	Poly	IF	1:100
p-38 MAPK	CST-9212	Mouse	Mono	WB	1:200
Phospho-p38	CST-9211	Mouse	Mono	WB	1:400
E-cadherin	BD-562869	Mouse	Mono	WB	1:2500
N-cadherin	Santacruz Sc-59987	Rabbit	Poly	WB	1:1000
β-Actin	BD612657	Mouse	Mono	WB	1:10,000
α-Mouse IgG-488	Sigma F8771	Goat	Poly	IF	1:200
α-Rabbit IgG-488	Sigma F9887	Goat	Poly	IF	1:200
α-Mouse IgG-555	Sigma SAB4600299	Goat	Poly	IF	1:200
α-Rabbit IgG-555	Sigma SAB4600268	Goat	Poly	IF	1:200
α-Mouse IgG-HRP	JIM 115–035-003	Goat	Poly	WB	1:5000
α-Rabbit IgG-HRP	JIM 111–035-003	Goat	Poly	WB	1:5000

### Protein extraction and Western blotting

TO confirm the regulatory role of PARP1 in islet neogenesis, clusters from each group were harvested, and total protein was isolated. After quantifying the protein concentration in each group^19^, micrograms of protein were loaded on 10% polyacrylamide SDS gels at 100 V and 1 h at room temperature conditions. After protein separation on poly acrylamide gels, migrated proteins were transferred and immunoblotted onto nitrocellulose membrane before probing for essential endocrine pathway proteins as specific antibodies, as described in Table 2. Transcription factors like Nestin, E-Cadherin, Pdx-1, Ngn-3, P-p38, p-38MAPK, N-cadherin and PARP1 were probed as described earlier^30^. Extended uncropped image source data of immune blots for RNAi and PARPi protein profiling experiments along with corresponding ponceau blots to verify loading controls are submitted as supplementary data files (please refer to Supplementary Fig.-3–20).

### Semi-quantitative gene expression

The presence of islet markers to confirm islet differentiation was measured using the semiquantitative reverse transcriptase PCR method and normalized to the human beta-actin gene. Total RNA was isolated and extracted using TRIZOL (Sigma). One µg of total RNA was reversed transcribed to first strand cDNA using superscript™ one-step RT-PCT kit, as per manufacturing protocol (Thermo Scientific, USA). Following RT, semi-quantitative gene expression was amplified and visualized using gene-specific primers under optimal PCR conditions for human insulin, glucagon and beta-actin genes. Primer sequences and PCR conditions are shown in Table 3. Amplified PCR products were run on 1.5% agarose gels at a constant 100 V for 30 min using low molecular weight (25-700BP) DNA ladder (NEB).

**Table 3:** Summarizes details of RT-PCR primer sequences and PCR conditions for islet cell differentiation markers.

Gene ID	PRIMER SEQ-Forward	PRIMER SEQ-Reverse	Tm	Size (bp)
Insulin	GCCCAGGCTTTTGTCAAACA	CTCCCCACACACCAGGTAGAG	55	90
Glucagon	ATGAAGACCATTTACTTTGTGGCT	GGTGTTCATCAACCACTGCAC	58	243
β-Actin	GGACTGTTACTGAGCTGCGTT	CGCCTTCACCGTTCCAGTT	57	209

### Statistical analysis

Statistical Analysis was performed using graph-pad prism-6 software using ttest, and two-way ANOVA method, and p-values were calculated with > 95% confidence interval, taking mean ± SEM into consideration with each experiment repeated 3 times, independently.

### Ethics approval and consent to participate

This research finding has been approved by the institutional ethics committee of the MS University of Baroda, India and the University of Laval, Quebec, Canada.

## Supplementary Information

Supplementary Figures.

## Data Availability

All relevant data are presented within the manuscript and its supplementary information files. No other relevant data files are available and required to deposit in additional database.

## References

[1] Pitocco D, Tesauro M, Alessandro R, Ghirlanda G, Cardillo C (2013). Oxidative stress in diabetes: Implications for vascular and other complications. Int. J. Mol. Sci..

[2] Matveeva E (2016). Involvement of PARP1 in the regulation of alternative splicing. Cell Discov..

[3] Sethi GS, Dharwal V, Naura AS (2017). Poly(ADP-Ribose)Polymerase-1 in lung inflammatory disorders: A review. Front. Immunol..

[4] Spina-Purrello V, Patti D, Giuffrida-Stella AM, Nicoletti VG (2008). Parp and cell death or protection in rat primary astroglial cell cultures under LPS/IFNgamma induced proinflammatory conditions. Neurochem. Res..

[5] Vyas S, Chang P (2014). New PARP targets for cancer therapy. Nat. Rev. Cancer.

[6] Akiyama T (2001). Activation of Reg gene, a gene for insulin-producing beta -cell regeneration: Poly(ADP-ribose) polymerase binds Reg promoter and regulates the transcription by autopoly(ADP-ribosyl)ation. Proc. Natl. Acad. Sci. USA.

[7] Alemasova EE, Lavrik OI (2019). Poly(ADP-ribosyl)ation by PARP1: Reaction mechanism and regulatory proteins. Nucleic Acids Res..

[8] Le Rhun Y, Kirkland JB, Shah GM (1998). Cellular responses to DNA damage in the absence of Poly(ADP-ribose) polymerase. Biochem. Biophys. Res. Commun..

[9] Yelamos J (2006). PARP-2 deficiency affects the survival of CD4+CD8+ double-positive thymocytes. EMBO J..

[10] Krishnakumar R, Kraus WL (2010). The PARP side of the nucleus: Molecular actions, physiological outcomes, and clinical targets. Mol. Cell.

[11] Ray-Chaudhuri A, Nussenzweig A (2017). The multifaceted roles of PARP1 in DNA repair and chromatin remodelling. Nat. Rev. Mol. Cell Biol..

[12] Ba X, Garg NJ (2011). Signaling mechanism of poly(ADP-ribose) polymerase-1 (PARP-1) in inflammatory diseases. Am. J. Pathol..

[13] Gao F, Kwon SW, Zhao Y, Jin Y (2009). PARP1 poly(ADP-ribosyl)ates Sox2 to control Sox2 protein levels and FGF4 expression during embryonic stem cell differentiation. J. Biol. Chem..

[14] Yamamoto H, Uchigata Y, Okamoto H (1981). Streptozotocin and alloxan induce DNA strand breaks and poly(ADP-ribose) synthetase in pancreatic islets. Nature.

[15] Berger NA (2018). Opportunities for the repurposing of PARP inhibitors for the therapy of non-oncological diseases. Br. J. Pharmacol..

[16] Huang D, Kraus WL (2022). The expanding universe of PARP1-mediated molecular and therapeutic mechanisms. Mol. Cell.

[17] Ogino H (2007). Loss of Parp-1 affects gene expression profile in a genome-wide manner in ES cells and liver cells. BMC Genom..

[18] Klein T (2003). Nestin is expressed in vascular endothelial cells in the adult human pancreas. J. Histochem. Cytochem..

[19] Burkart V (1999). Mice lacking the poly(ADP-ribose) polymerase gene are resistant to pancreatic beta-cell destruction and diabetes development induced by streptozocin. Nat. Med..

[20] Pieper AA (1999). Poly(ADP-ribose) polymerase-deficient mice are protected from streptozotocin-induced diabetes. Proc. Natl. Acad. Sci. USA.

[21] Yonemura Y (1984). Amelioration of diabetes mellitus in partially depancreatized rats by poly(ADP-ribose) synthetase inhibitors. Evidence of islet B-cell regeneration. Diabetes.

[22] Gong L (2012). Poly (ADP-ribose) transferase/polymerase-1-deficient mice resistant to age-dependent decrease in beta-cell proliferation. Mol. Med..

[23] Uchigata Y, Yamamoto H, Kawamura A, Okamoto H (1982). Protection by superoxide dismutase, catalase, and poly(ADP-ribose) synthetase inhibitors against alloxan- and streptozotocin-induced islet DNA strand breaks and against the inhibition of proinsulin synthesis. J. Biol. Chem..

[24] Brownlee M (2005). The pathobiology of diabetic complications: A unifying mechanism. Diabetes.

[25] Garcia-Soriano F (2001). Diabetic endothelial dysfunction: The role of poly(ADP-ribose) polymerase activation. Nat. Med..

[26] Obrosova IG (2004). Role of poly(ADP-ribose) polymerase activation in diabetic neuropathy. Diabetes.

[27] Zheng L, Szabo C, Kern TS (2004). Poly(ADP-ribose) polymerase is involved in the development of diabetic retinopathy via regulation of nuclear factor-kappaB. Diabetes.

[28] Srivastava A, Dadheech N, Vakani M, Gupta S (2019). Pancreatic resident endocrine progenitors demonstrate high islet neogenic fidelity and committed homing towards diabetic mice pancreas. J. Cell Physiol..

[29] Ye DZ, Tai MH, Linning KD, Szabo C, Olson LK (2006). MafA expression and insulin promoter activity are induced by nicotinamide and related compounds in INS-1 pancreatic beta-cells. Diabetes.

[30] Dadheech N (2015). Swertisin an anti-diabetic compound facilitate islet neogenesis from pancreatic stem/progenitor cells via p-38 MAP kinase-SMAD pathway: An in-vitro and in-vivo study. PLoS ONE.

[31] Dadheech N (2020). Direct lineage tracing reveals Activin-a potential for improved pancreatic homing of bone marrow mesenchymal stem cells and efficient ss-cell regeneration in vivo. Stem Cell Res. Ther..

[32] Hardikar AA, Marcus-Samuels B, Geras-Raaka E, Raaka BM, Gershengorn MC (2003). Human pancreatic precursor cells secrete FGF2 to stimulate clustering into hormone-expressing islet-like cell aggregates. Proc. Natl. Acad. Sci. USA.

[33] Gupta S, Dadheech N, Singh A, Soni S, Bhonde R (2010). Enicostemma littorale: A new therapeutic target for islet neogenesis. Int. J. Integr. Biol..

[34] Rouleau M, Patel A, Hendzel MJ, Kaufmann SH, Poirier GG (2010). PARP inhibition: PARP1 and beyond. Nat. Rev. Cancer.

[35] Schreiber V, Dantzer F, Ame JC, de Murcia G (2006). Poly(ADP-ribose): Novel functions for an old molecule. Nat. Rev. Mol. Cell Biol..

[36] Ji Y, Tulin AV (2010). The roles of PARP1 in gene control and cell differentiation. Curr. Opin. Genet. Dev..

[37] Wang ZQ (1995). Mice lacking ADPRT and poly(ADP-ribosyl)ation develop normally but are susceptible to skin disease. Genes Dev..

