# Supplementary material for: Role of poly(ADP-ribose) polymerase-1 in regulating human islet cell differentiation
Source: Sci Rep. 2022 Dec 13;12:21496. doi: 10.1038/s41598-022-25405-w (PMC9747708; doi:10.1038/s41598-022-25405-w)
Supplement: Supplementary file 1 — Supplementary Figures. [file 41598_2022_25405_MOESM1_ESM.pdf]

Confirmation of islet differentiation in human PANC-1 cells with activin-A

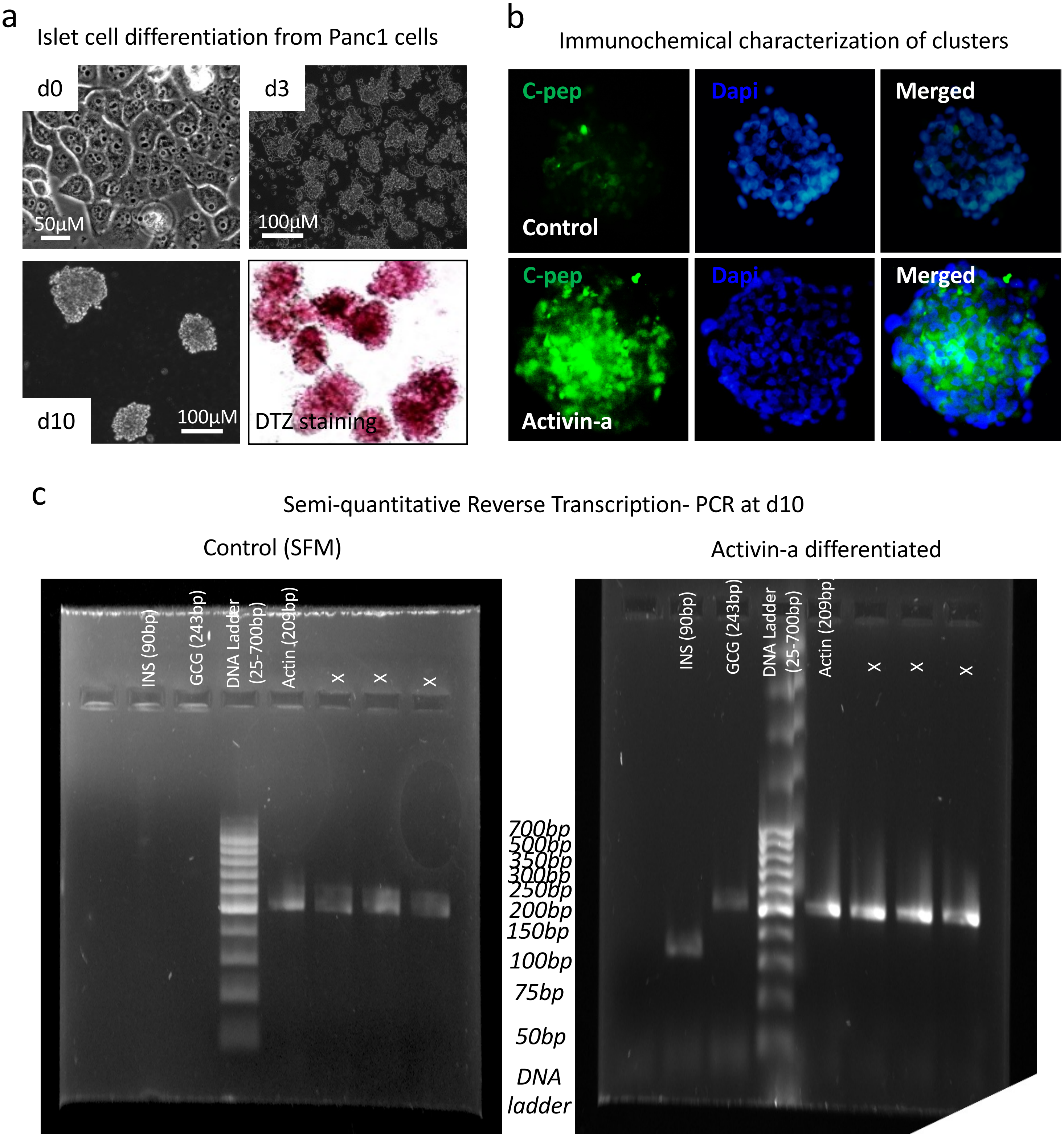

Supplementary Figure 1. In-vitro islet differentiation of human PANC-1 cells using activin-A in 10-day differentiation scheme. **(a)** Phase contrast microscopic images of PANC-1 cells treated with activin-A at day 0, 3rd and 10th and color image for DTZ stain (brick-red). **(b)** immunofluorescent images for human C-peptide (green) in islet clusters from SFM control and activin-A at 10th day. Dapi (blue) represents nuclei **(c)** Semi quantitative reverse transcriptase-PCR gel to detect insulin (90bp) and glucagon (243bp) transcripts in activin-A differentiated PANC-1 cell clusters at day 10th. Beta-actin (209bp) is used as internal control.

Confirmation of differentiation in PJ34 and activin-A treated PANC-1 cells with short (1-9h) time dependent immunoblotting study

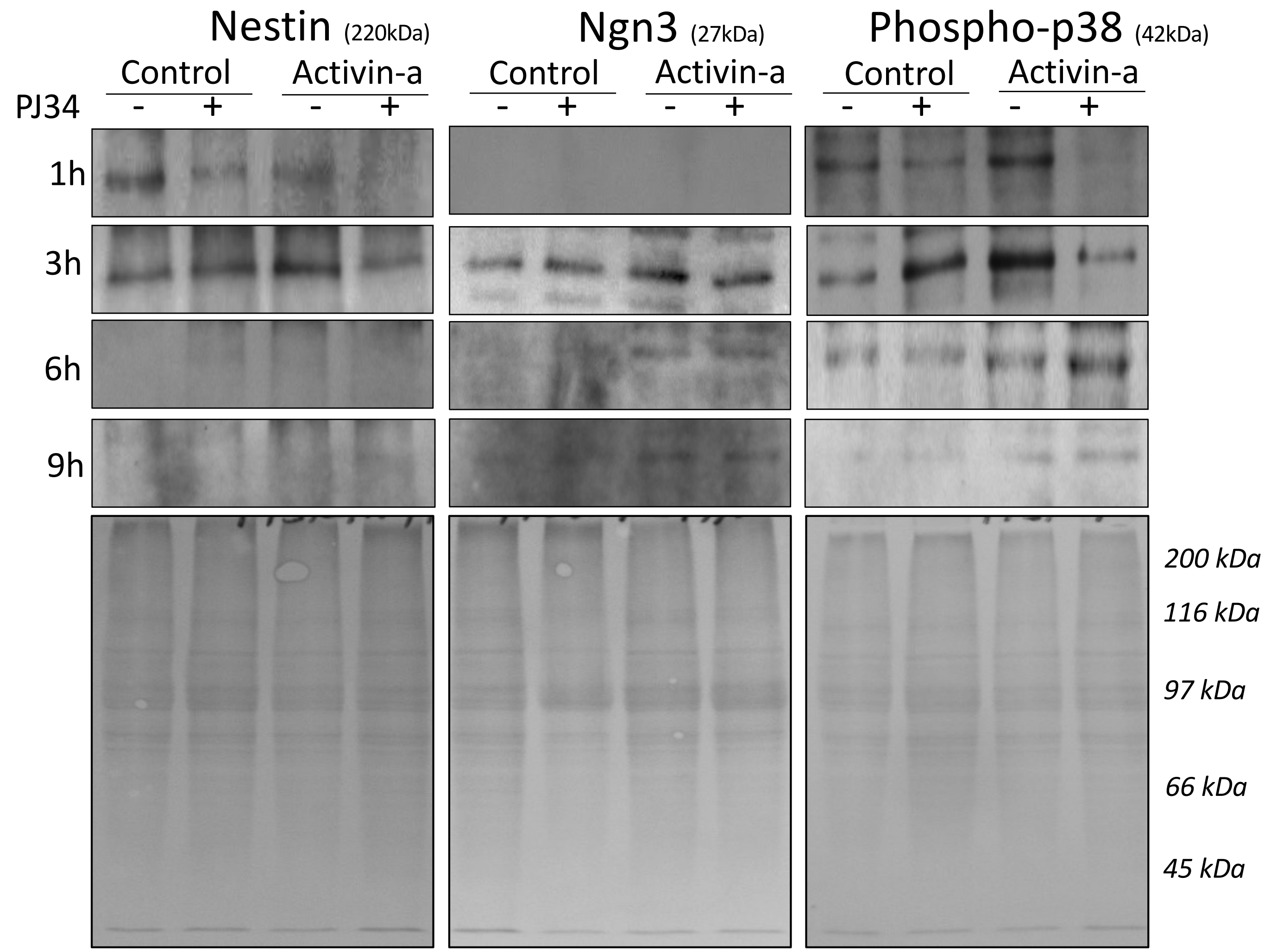

Supplementary Figure 2. Short-term time dependent study (1-9 hours) using immunoblot profiling of key endocrine differentiation markers from activin-a and SFM control differentiated islet cells treated in combination with PARPi- PJ34. Ponceau blots are shown as a loading control with a molecular weight marker. Full uncropped original sourced images for each immunoblots and corresponding ponceau blots are shown in supplementary figures 3-5.

Uncropped immunoblot images for supplementary figure-2

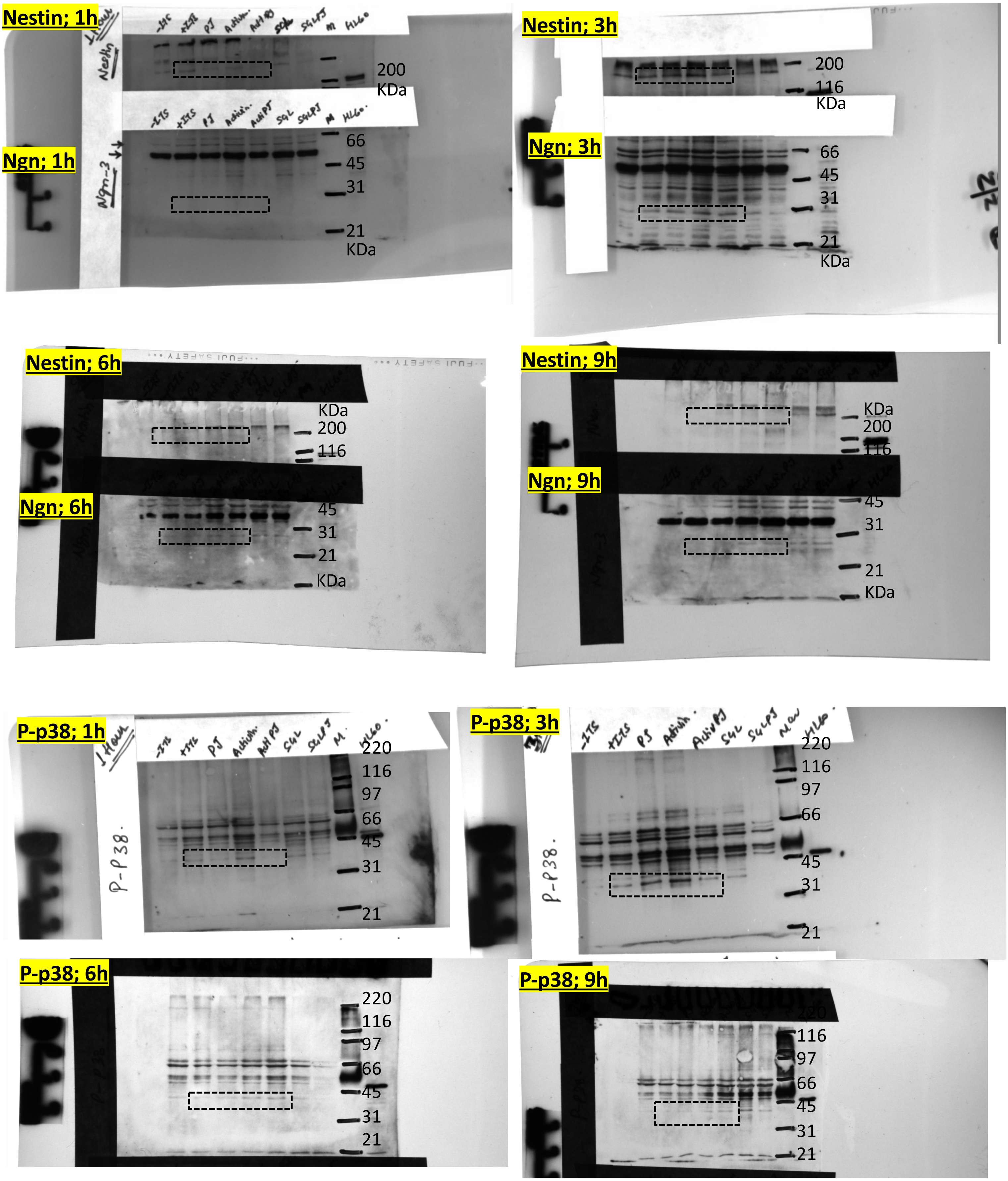

Supplementary Figure 3. Uncropped full-legth sourced immunoblot images for short [1–9-hour] time dependent study for Nestin, Ngn3 and P-p38 proteins in human PANC-1 cells differentiated clusters with SFM control and activin-A in combination with PJ34.

Uncropped ponceau stained images for supplementary figure-2

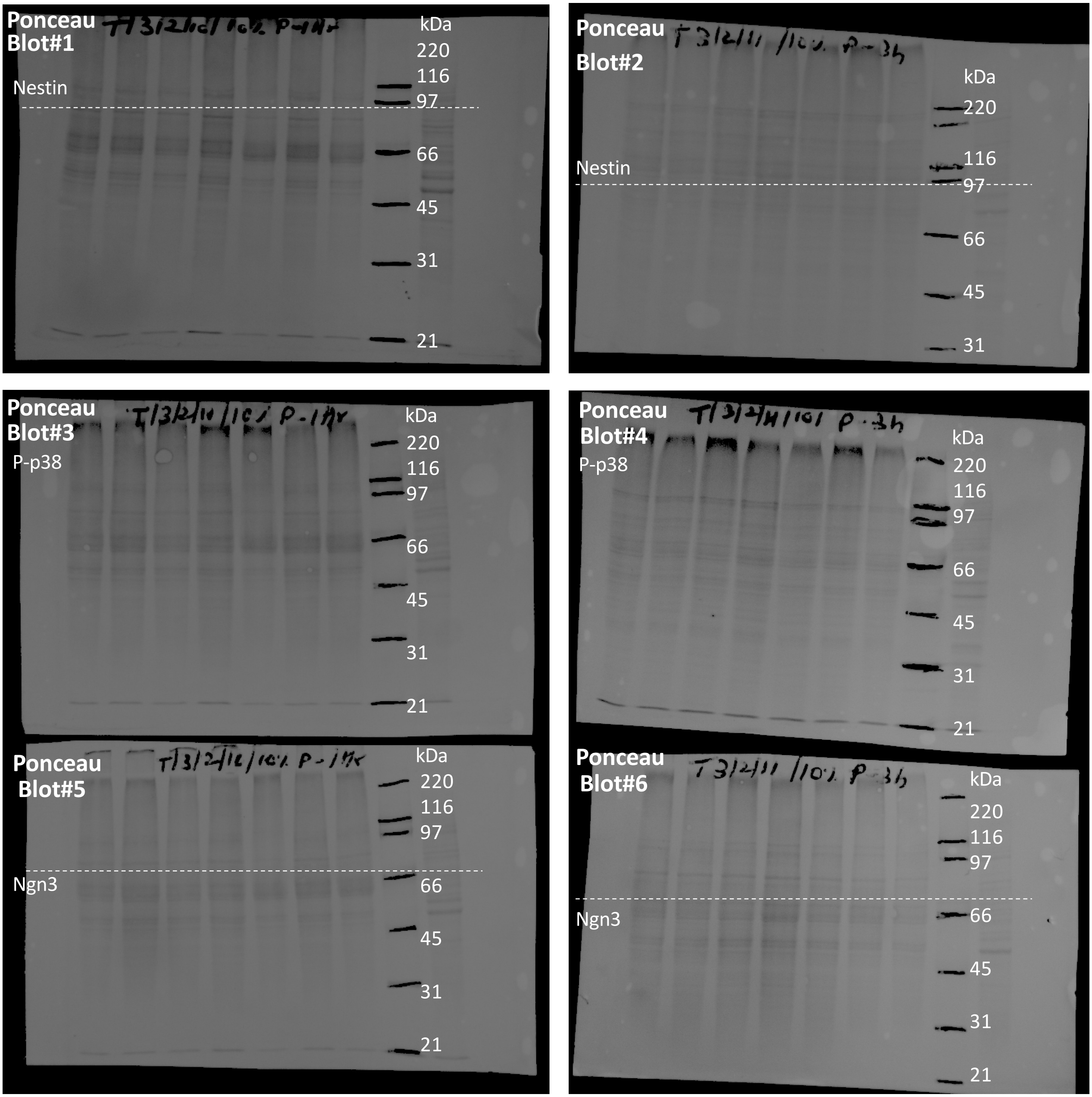

Supplementary Figure 4. Corresponding uncropped ponceau stained blots to show the original source data for 1–3-hours time course as loading control for Nestin, Ngn3 and P-p38 proteins in PANC-1 differentiated clusters with SFM control and activin-A in combination with PJ34. Dashed white lines represent membrane cut at desired molecular weight to allow for multiple protein probing using the same blot.

Uncropped ponceau stained images for supplementary figure-2

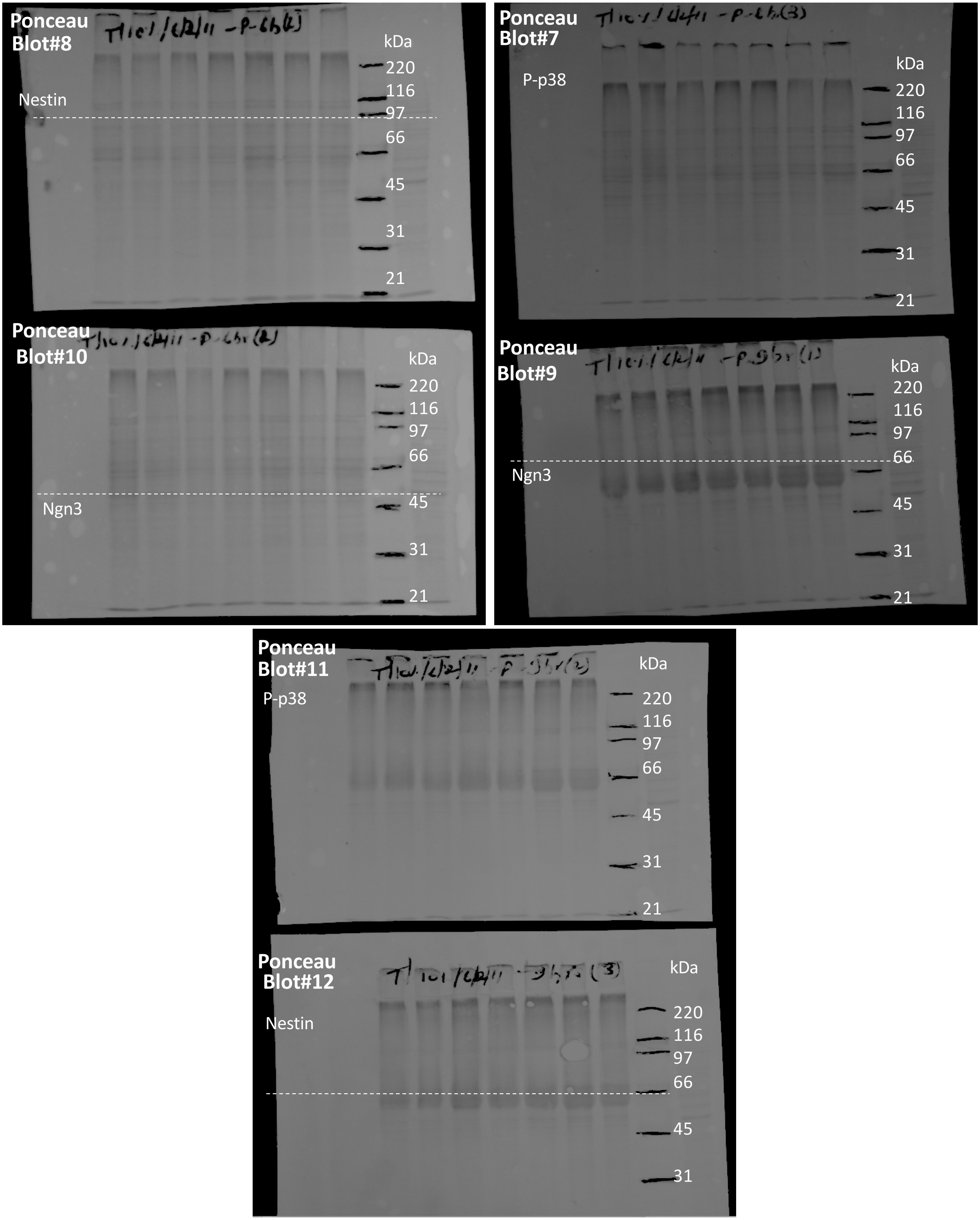

Supplementary Figure 5. Corresponding uncropped ponceau stained blots to show the original source data for 6-9 hours time course as loading control for Nestin, Ngn3 and P-p38 proteins in PANC-1 differentiated clusters with SFM control and activin-A in combination with PJ34. Dashed white lines represent membrane cut at desired molecular weight to allow for multiple protein probing using the same blot.

Schematic representation of shRNA construct and confirmation for PARP1 knockdown in PANC-1 cells

a

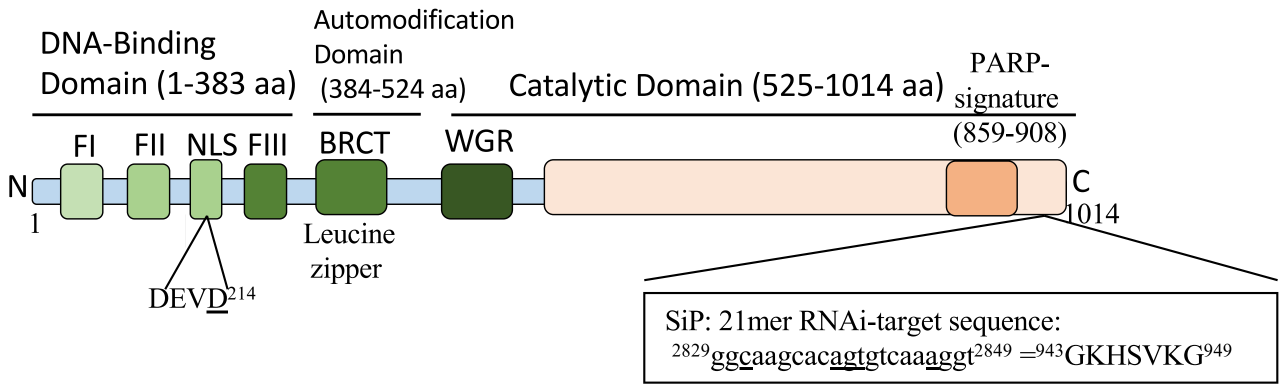

Supplementary Figure 6a. Schematic representation of shRNA construct and sequence location for SiP912-21 mer RNAi target used for PARP-1 depletion in human PANC-1 cells. The illustration is created using Microsoft 365- Version 16.64 (https://www.office.com/).

b

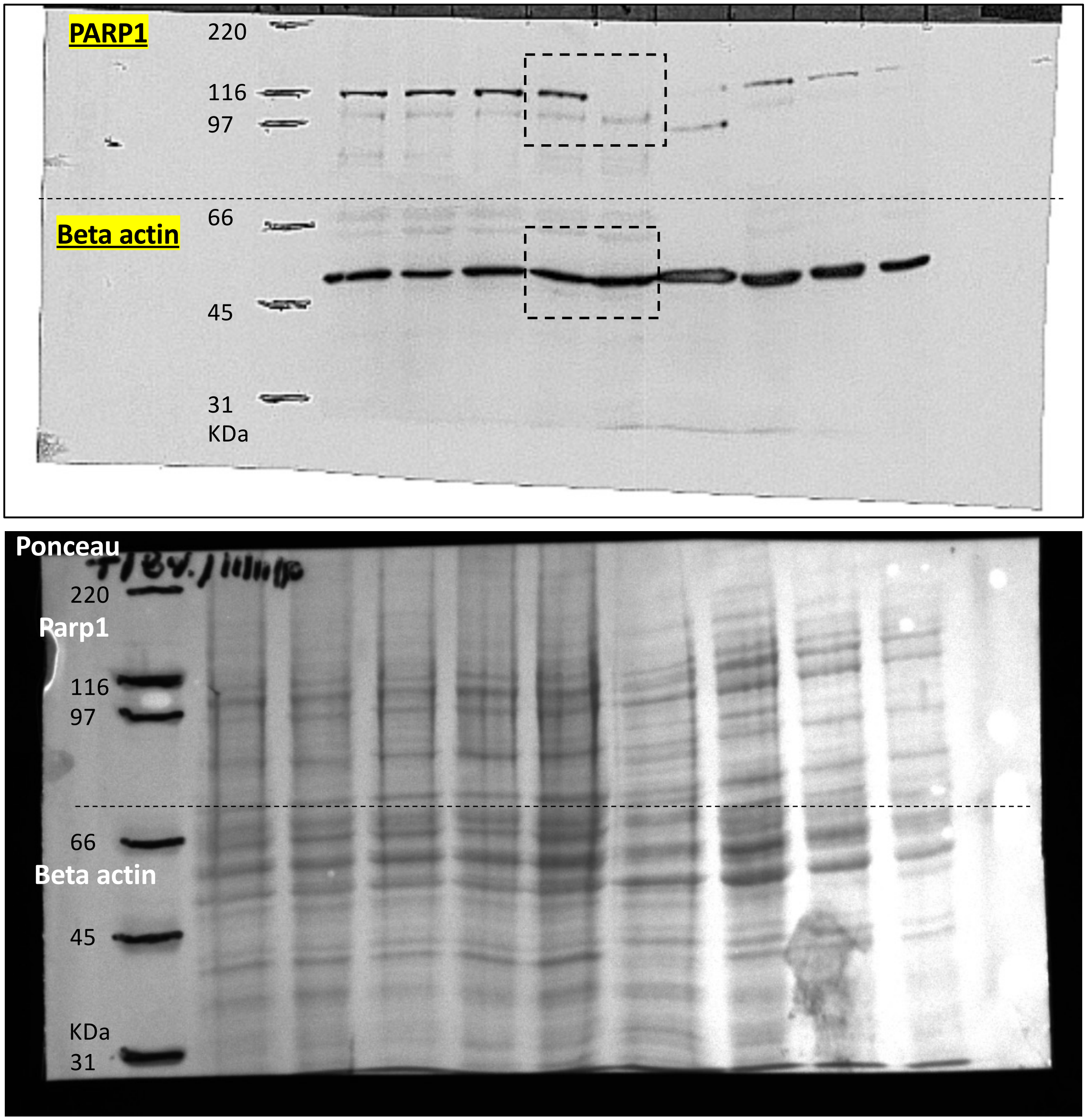

Supplementary Figure 6b. Uncropped full length immunoblot image to show original source data for PARP1 silencing in human PANC-1 cells using RNAi constructs for control-U6 and PARP1 deplete-SiP cells. Corresponding ponceau blots shown as loading control with a molecular weight marker.

Uncropped immunoblot images for islet differentiation markers in PARP1 U6 and SiP cells in Fig4a (d10)

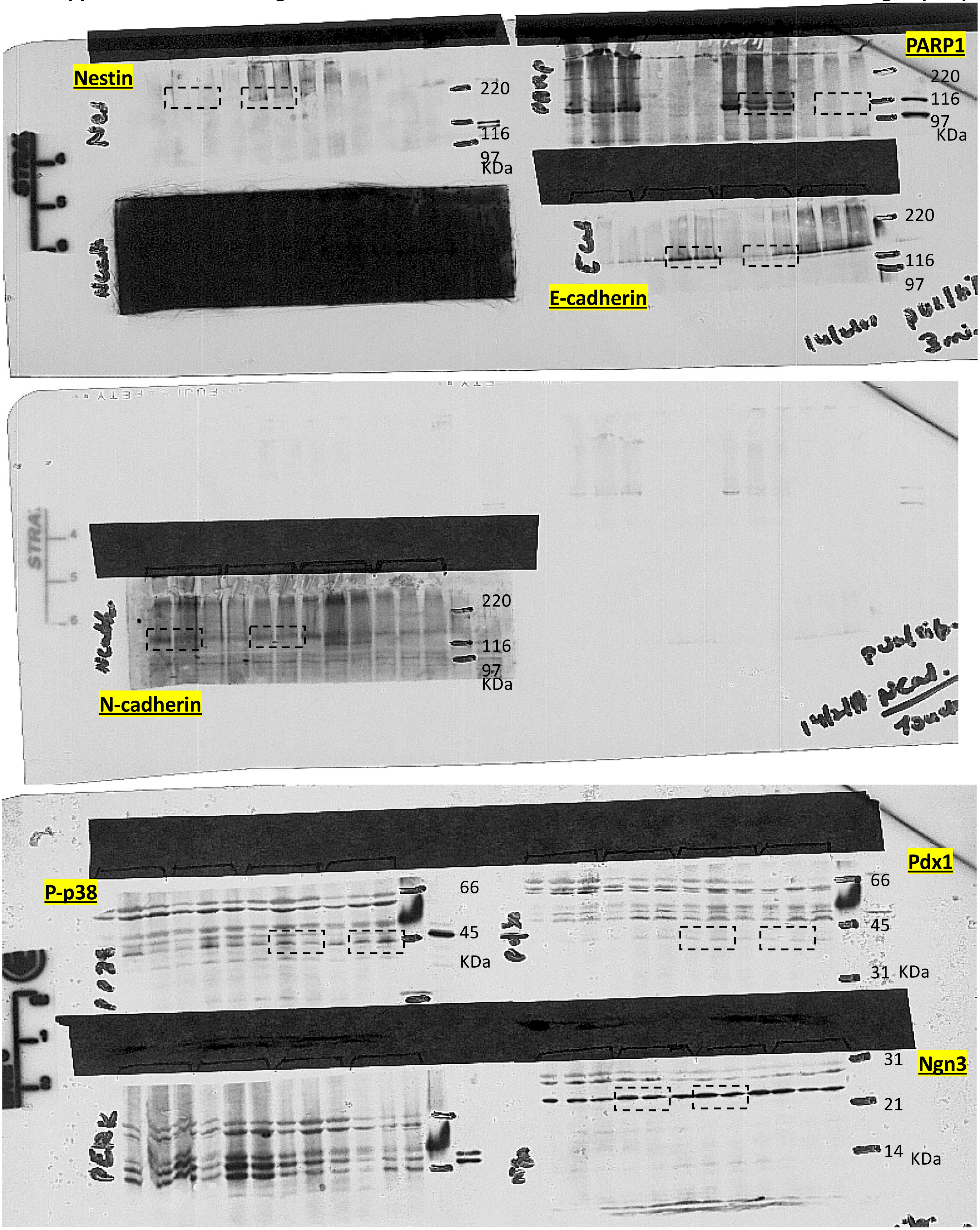

Supplementary Figure 7. Uncropped full-length immunoblot images to show original source data for PARP1 and endocrine cell differentiation proteins (Nestin, E-Cadherin, N-Cadherin, P-p38, PDX-1 and Ngn3) using RNAi produced U6 and SiP-derived clusters differentiated with SFM control and activin-A.

Uncropped ponceau stain images of immunoblots for PARP1 U6 and SiP differentiated cells in Fig4a (d10)

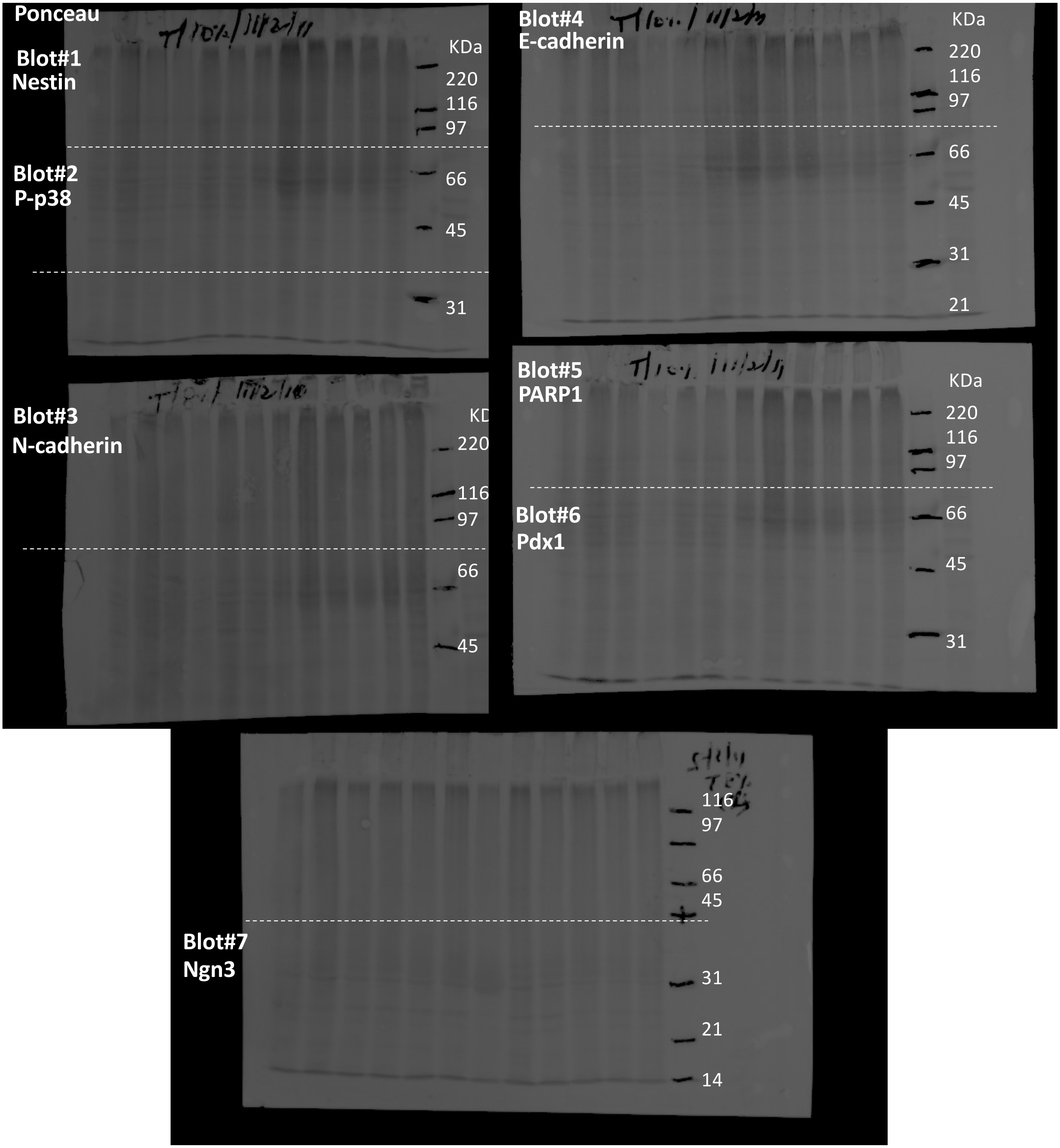

Supplementary Figure 8. Corresponding uncropped ponceau stained blots to show the original source data for PARP1 and endocrine cell differentiation proteins (Nestin, E-Cadherin, N-Cadherin, P-p38, PDX-1 and Ngn3) probed using lysates from RNAi produced U6 and Sip-differentiated islet cells with SFM control and activin-A as shown in supplementary figure 7. Dashed white lines represent membrane cut at molecular weight to allow for multiple protein probing using the same blot.

Uncropped immunoblot images for PARP1 protein in PANC-1 cells with and without PJ34 shown in Fig5b

a

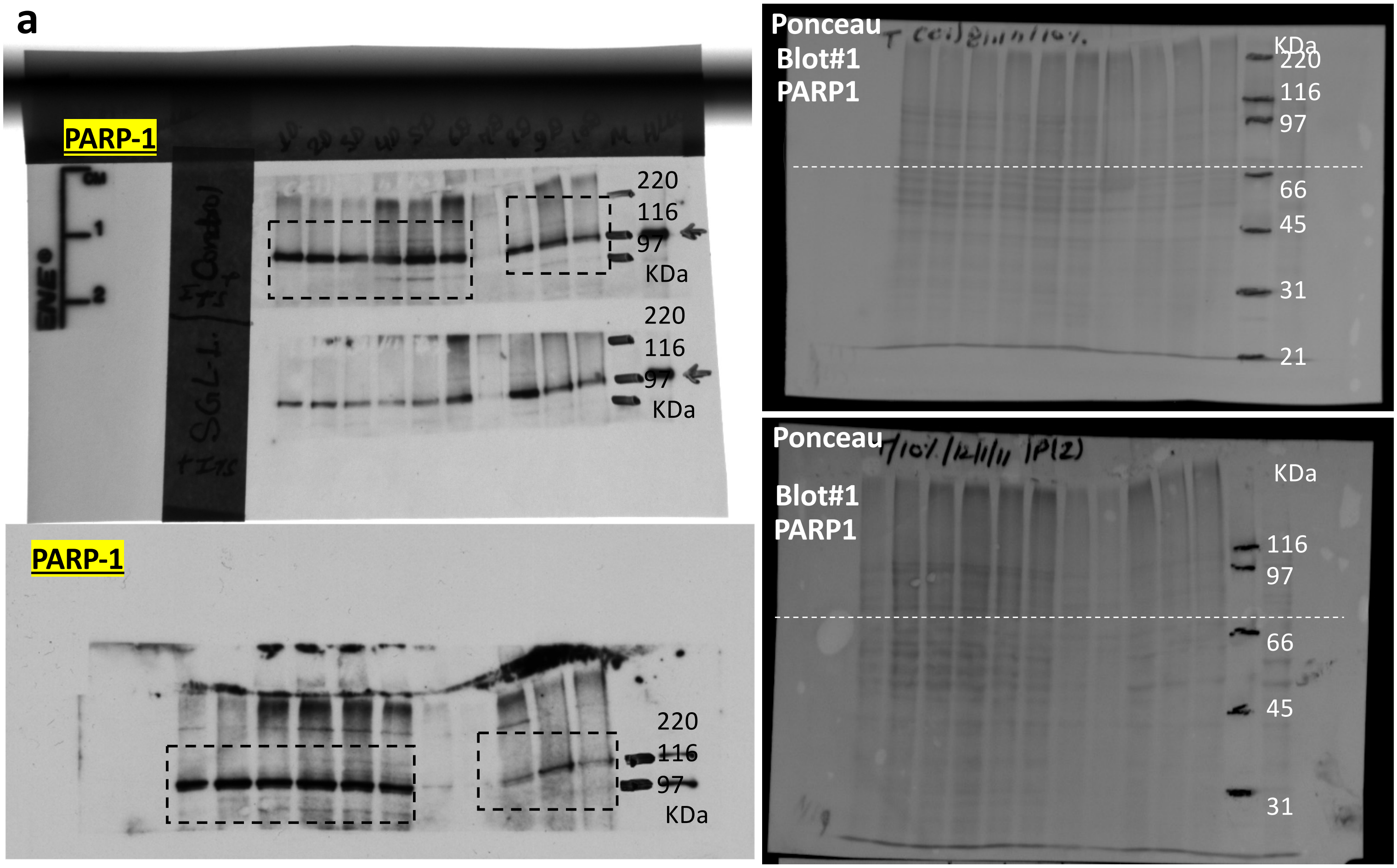

Supplementary Figure 9a. Uncropped full length immunoblot images to show original source data for PARP1 protein in PANC-1 cells with and without PJ34. Corresponding ponceau blots show the loading control with a molecular weight marker. Lines represent membrane cut.

Uncropped immunoblot images for PARP1 in PANC-1 differentiated clusters at d10 shown in fig6a

b

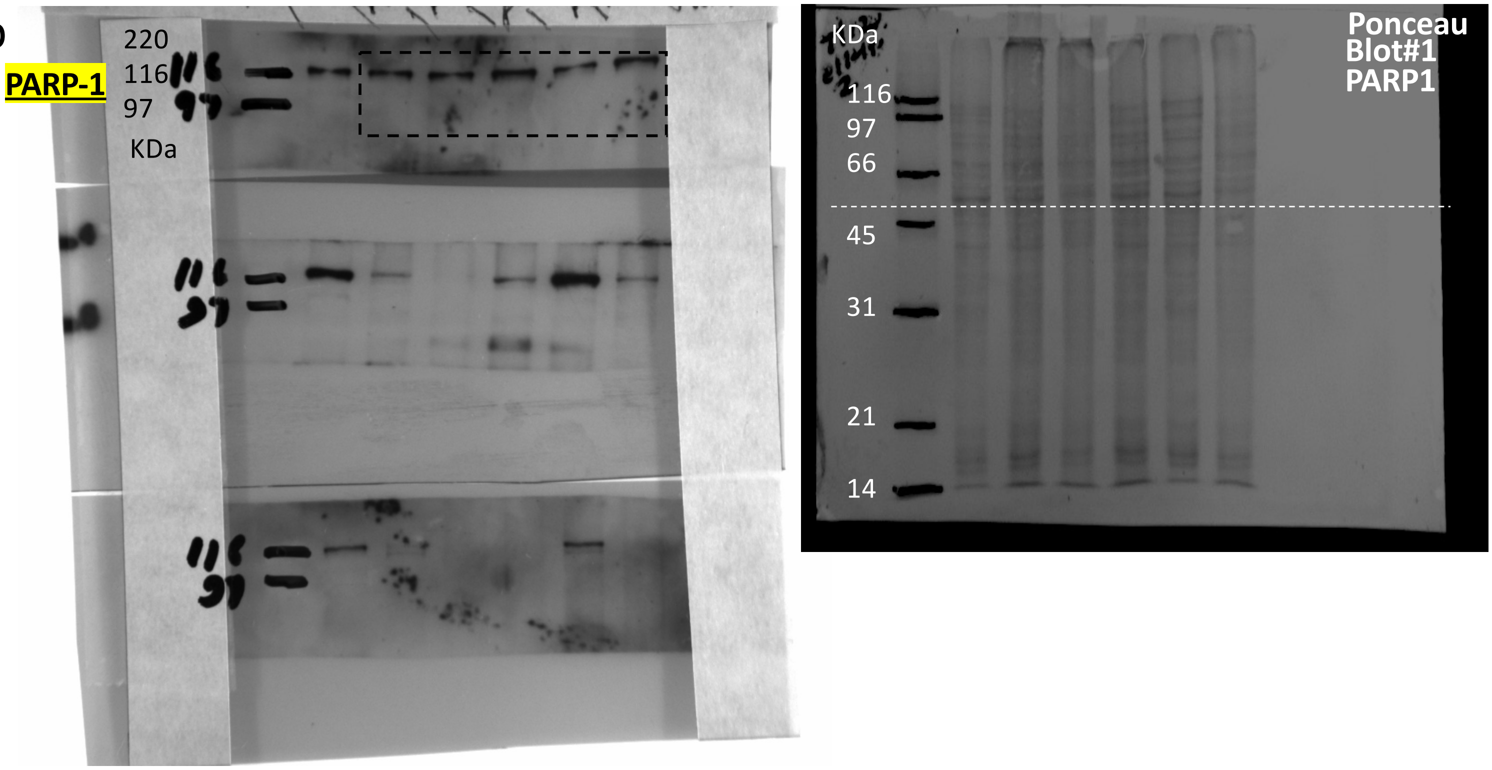

Supplementary Figure 9b. Uncropped full length immunoblot images to show original source data for PARP1 protein in PANC-1 islet clusters after 10 day of differentiation with SFM control and activin-A in presence or absence of PJ34. Corresponding ponceau blots show the loading control with a molecular weight marker. Lines represent membrane cut.

Uncropped immunoblot images for differentiation markers in PANC-1 clusters at d10 shown in fig6a

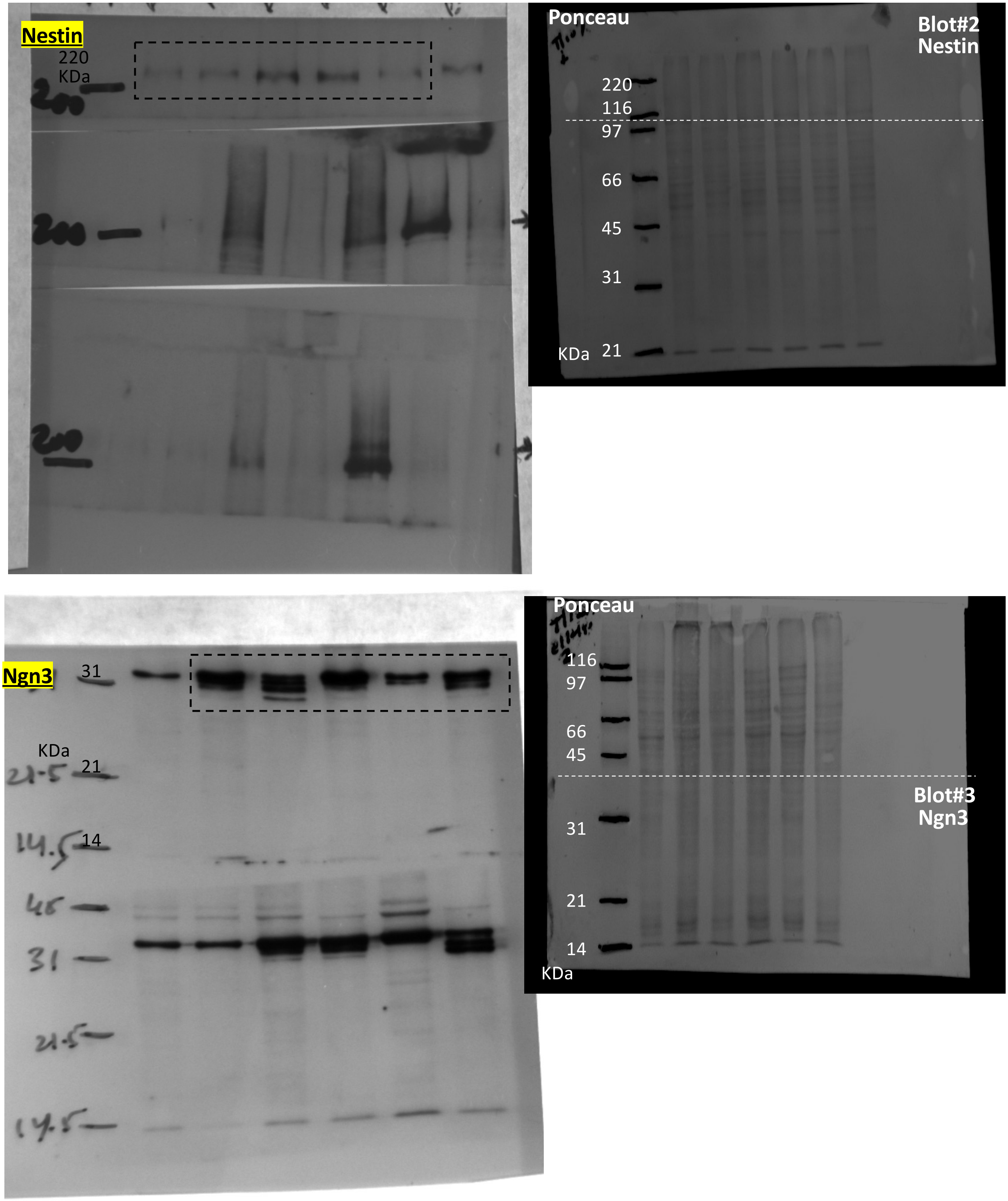

Supplementary Figure 10. Uncropped full-length immunoblot images to show original source data for Nestin and Ngn3 proteins in PANC-1 cells differentiated at day 10th with SFM control and activin-A in presence or absence of PJ34. Corresponding ponceau blots show the loading control with a molecular weight marker. Lines represent membrane cut.

Uncropped immunoblot images for differentiation markers in PANC-1 clusters at d10 shown in fig6a

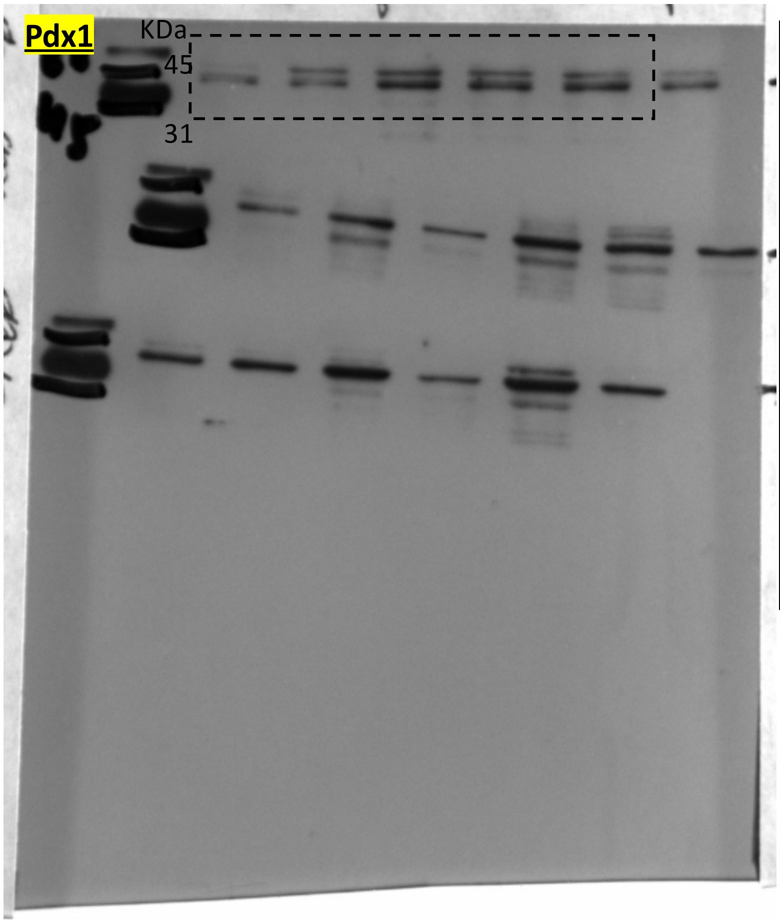

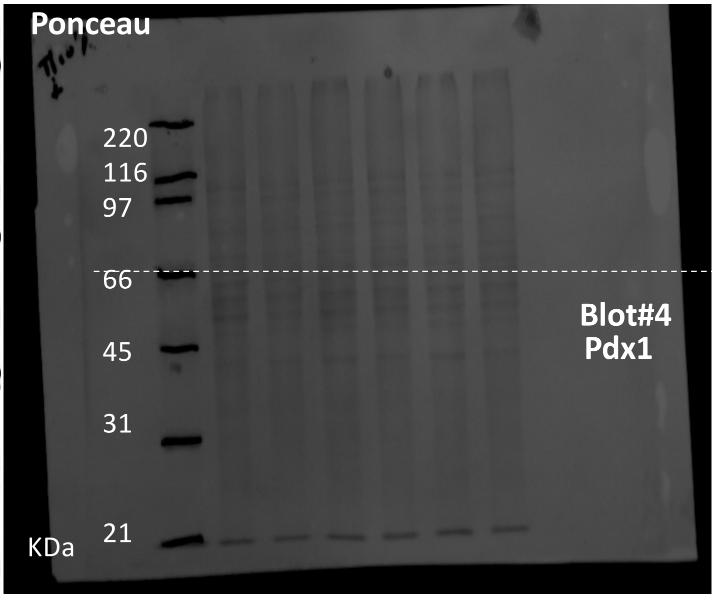

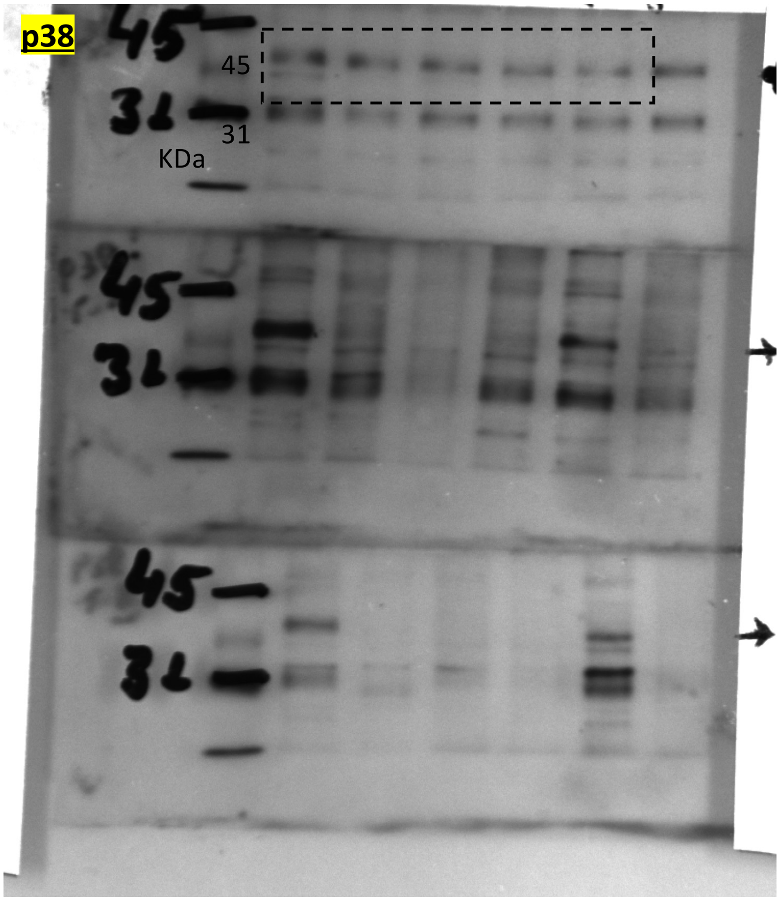

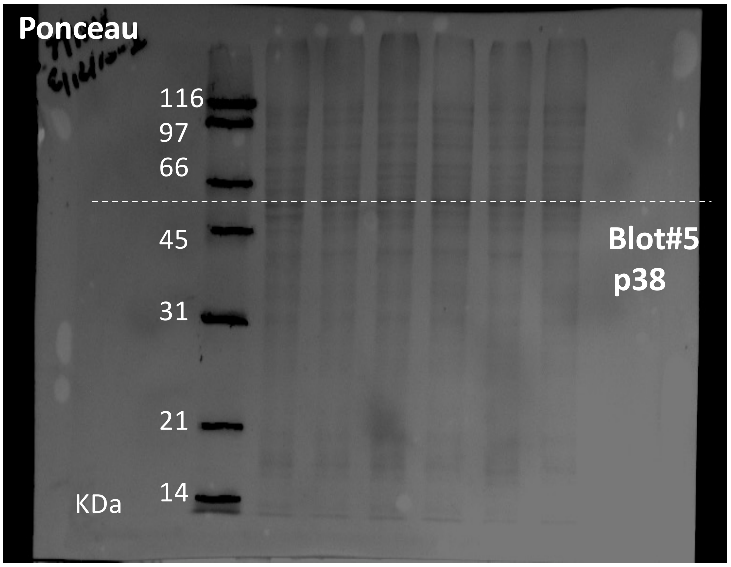

Supplementary Figure 11. Uncropped full-length immunoblot images to show original source data for Pdx1 and p-38 proteins in PANC-1 cells differentiated at day 10th with SFM control and activin-A in presence or absence of PJ34. Corresponding ponceau blots show the loading control with a molecular weight marker. Lines represent membrane cut.

Uncropped immunoblot images for differentiation markers in PANC-1 clusters at d10 shown in fig6a

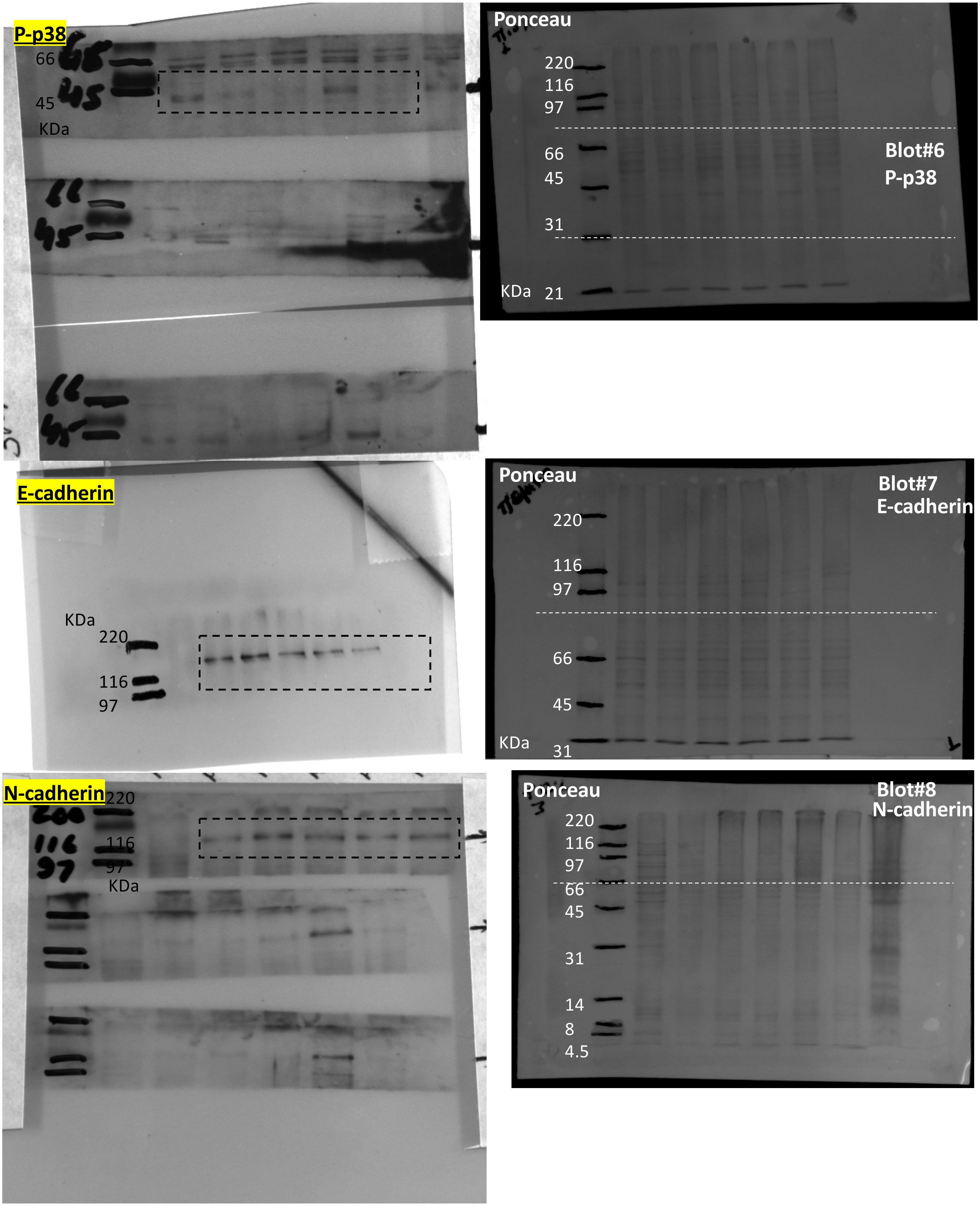

Supplementary Figure 12. Uncropped full-length immunoblot images to show original source data for P-p38, E-cadherin and N-cadherin proteins in PANC-1 cells differentiated at day 10th with SFM control and activin-A in presence or absence of PJ34. Corresponding ponceau blots show the loading control with a molecular weight marker. Lines represent membrane cut.

Uncropped immunoblot images for differentiation markers in PANC-1 clusters from time course study (d1-6) shown in fig6c

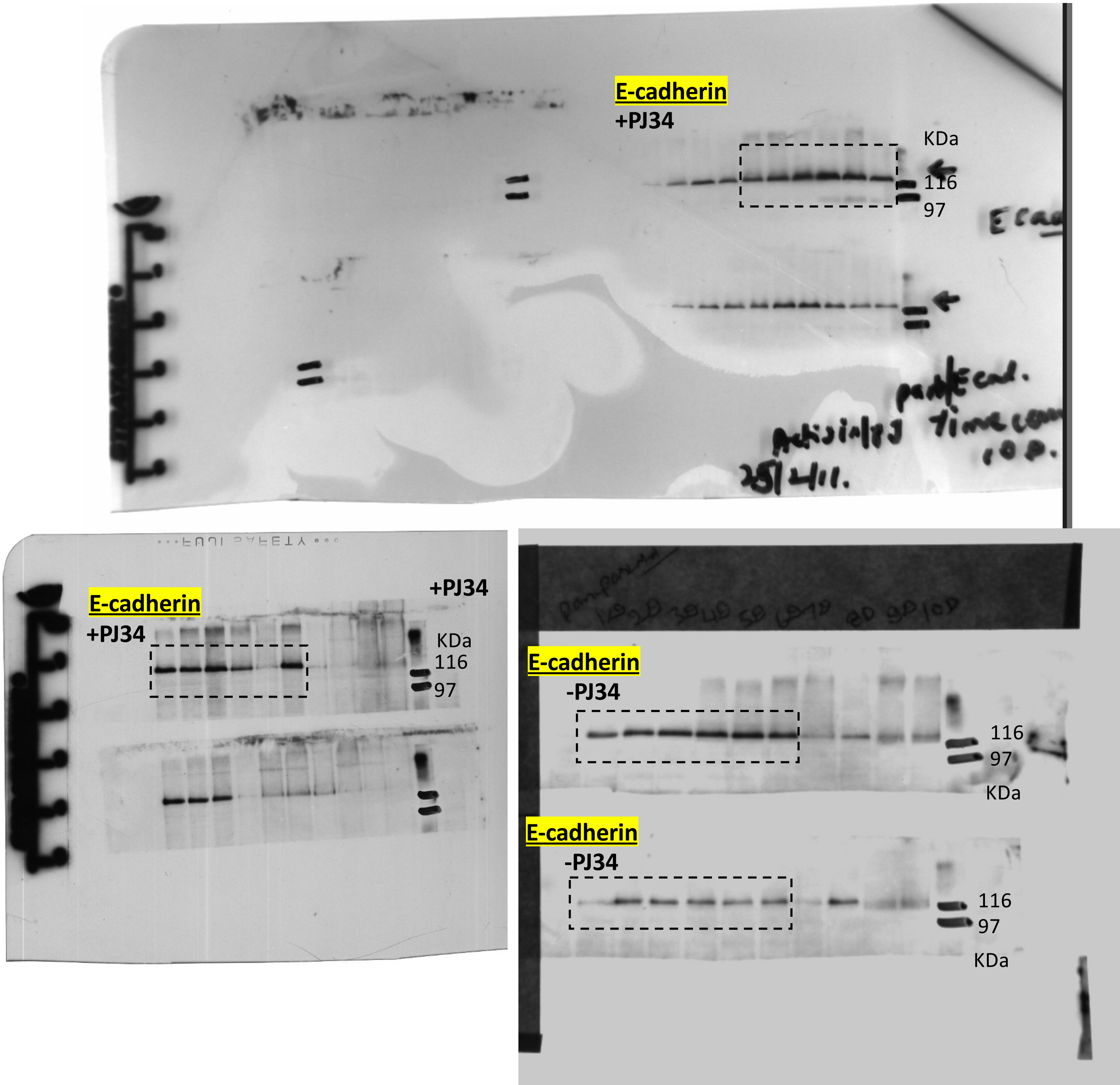

Supplementary Figure 13. Uncropped full- length immunoblot images from 10-day time course study to show original source data for E-cadherin protein in PANC-1 differentiated clusters with SFM control and activin-A in presence or absence of PJ34 as shown in fig 6c.

Uncropped ponceau blot images of differentiation markers in time course study (d1-6) shown in fig6c

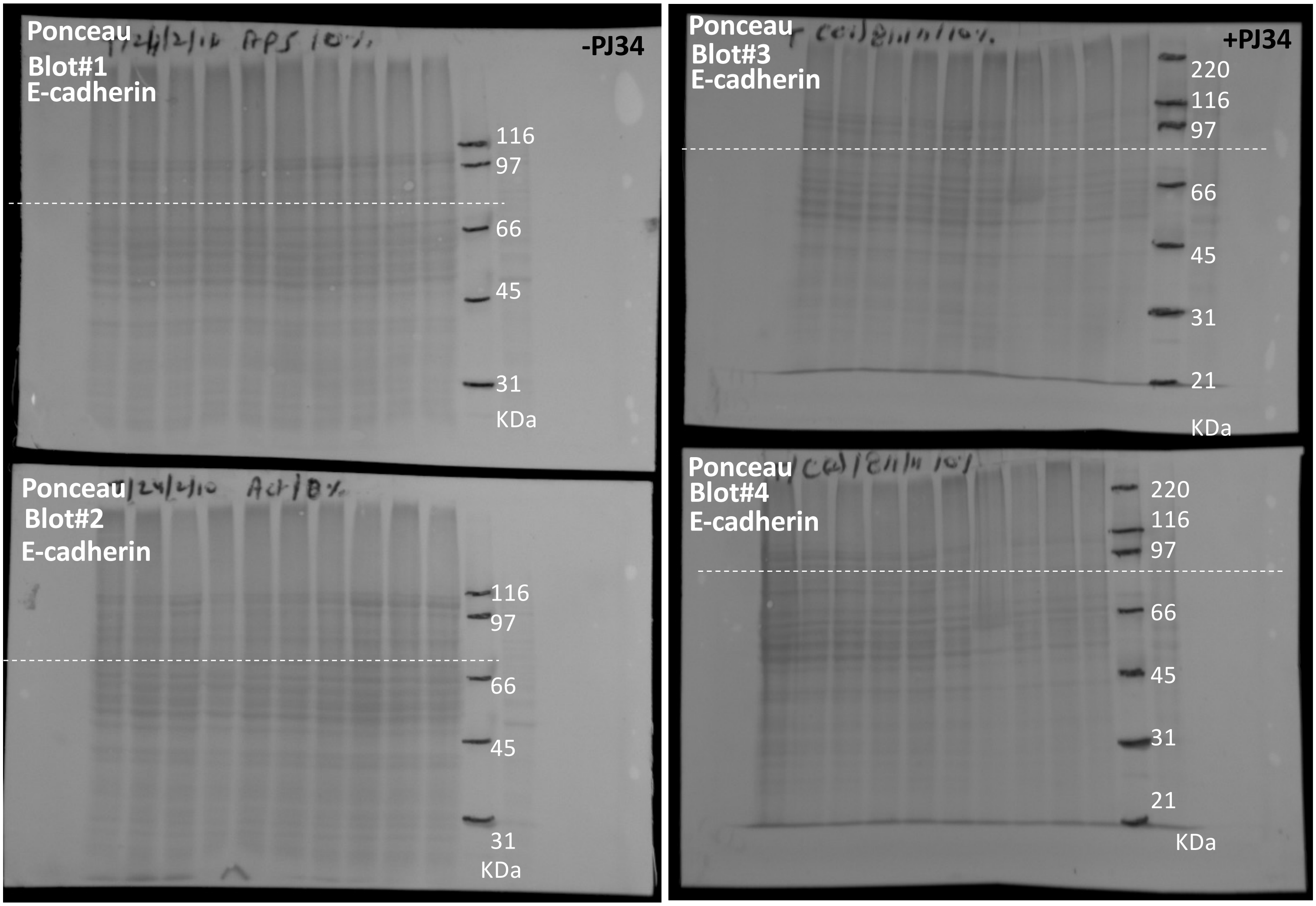

Supplementary Figure 14. Corresponding uncropped ponceau stained blots to show the original source data for E-cadherin protein in lysates from PANC-1 differentiated clusters with SFM control and activin-A in presence or absence of PJ34 as shown in supplementary fig 13. Dashed white lines represent membrane cut at molecular weight to allow for multiple protein probing using the same blot.

Uncropped immunoblot images for differentiation markers in PANC-1 clusters from time course study (d1-6) shown in fig6c

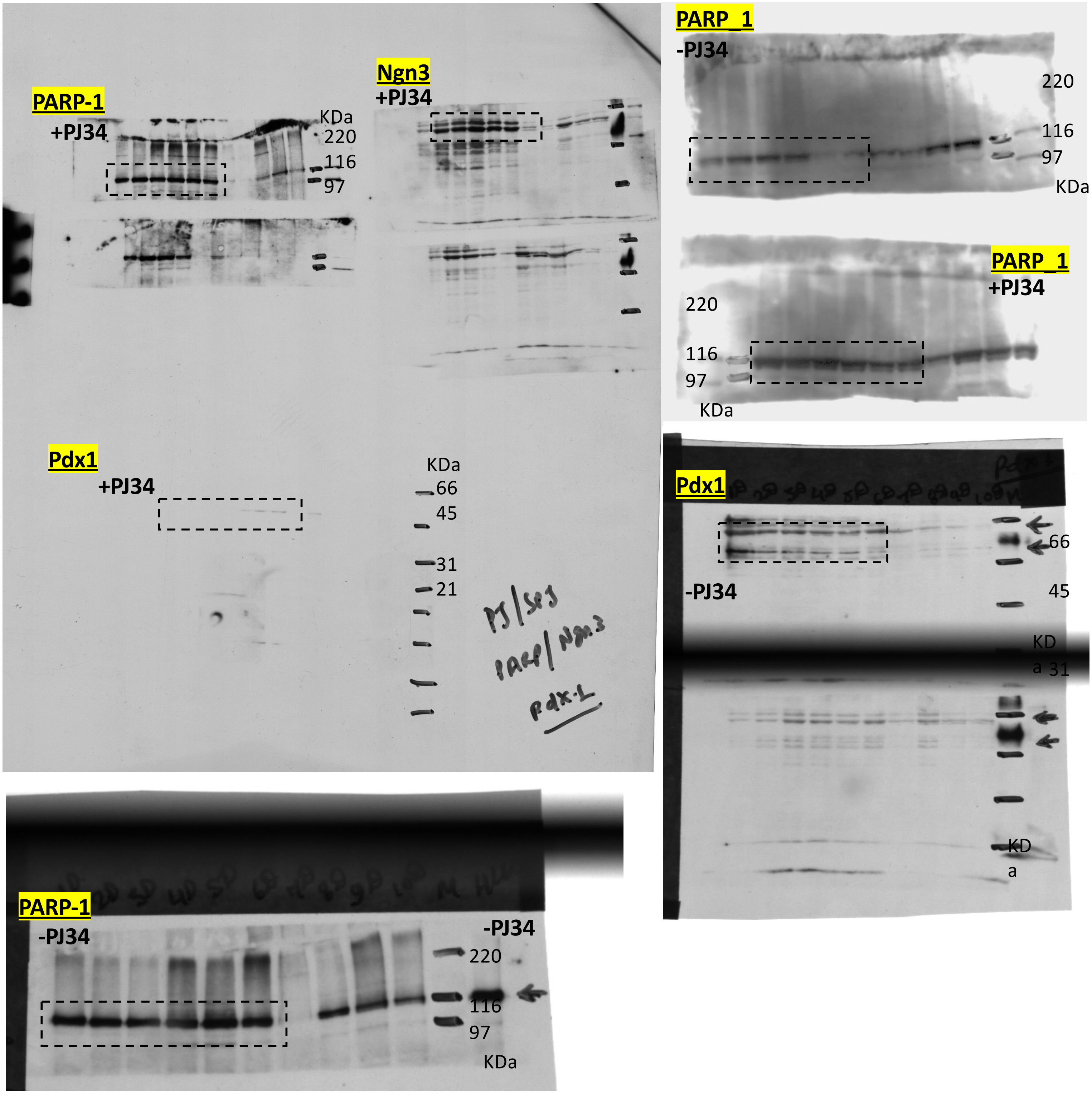

Supplementary Figure 15. Uncropped full-length immunoblot images from 10-day time course study to show original source data for PARP1 and Pdx1 proteins in PANC-1 differentiated clusters with SFM control and activin-A in presence or absence of PJ34 as shown in fig 6c.

Uncropped ponceau blot images of differentiation markers in time course study (d1-6) shown in fig6c

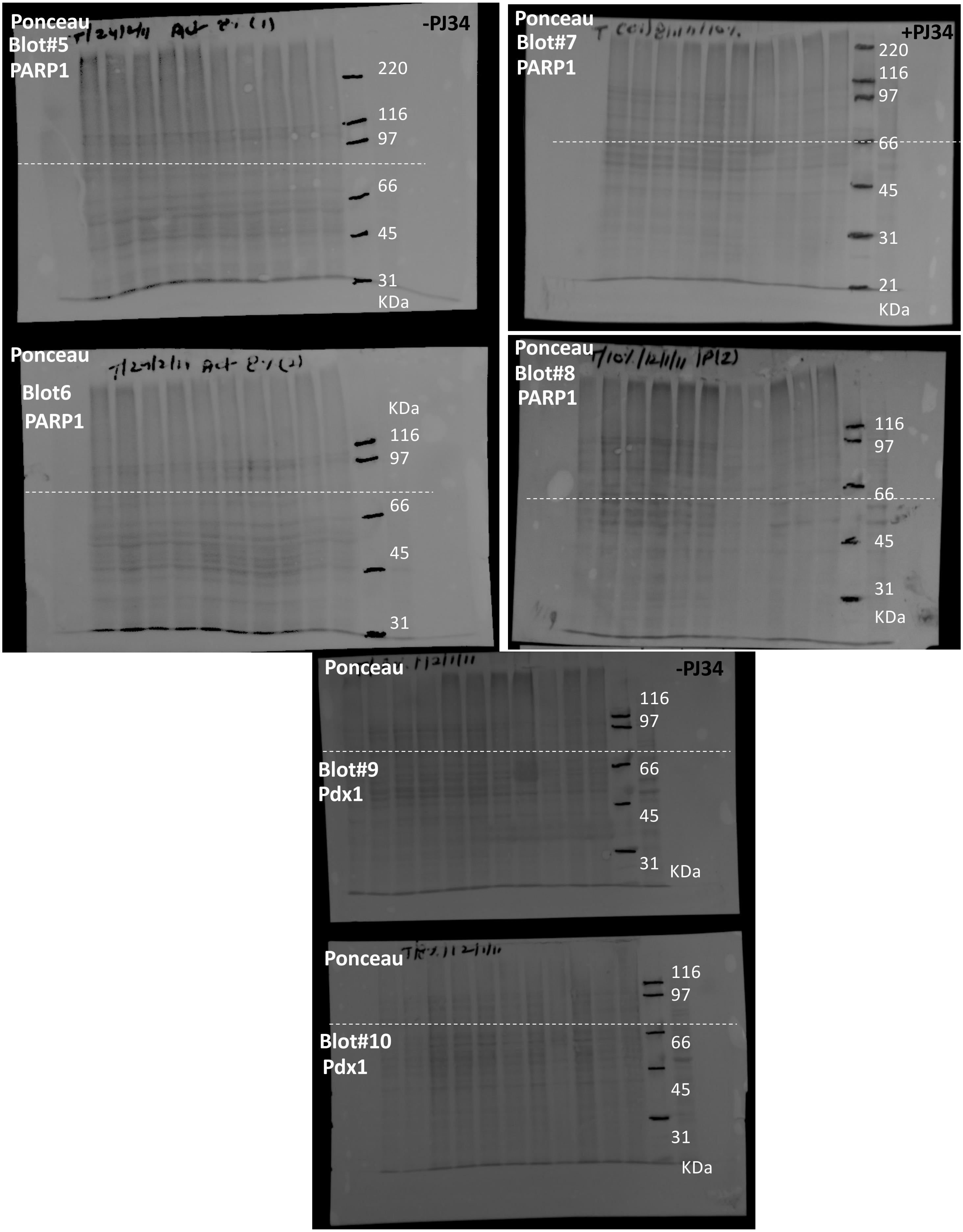

Supplementary Figure 16. Corresponding uncropped ponceau stained blots to show the original source data for PARP1 and Pdx1 protein in lysates from PANC-1 differentiated clusters with SFM control and activin-A in presence or absence of PJ34 as shown in supplementary fig 15. Dashed white lines represent membrane cut at molecular weight to allow for multiple protein probing using the same blot.

Uncropped immunoblot images for differentiation markers in PANC-1 clusters from time course study (d1-6) shown in fig6c

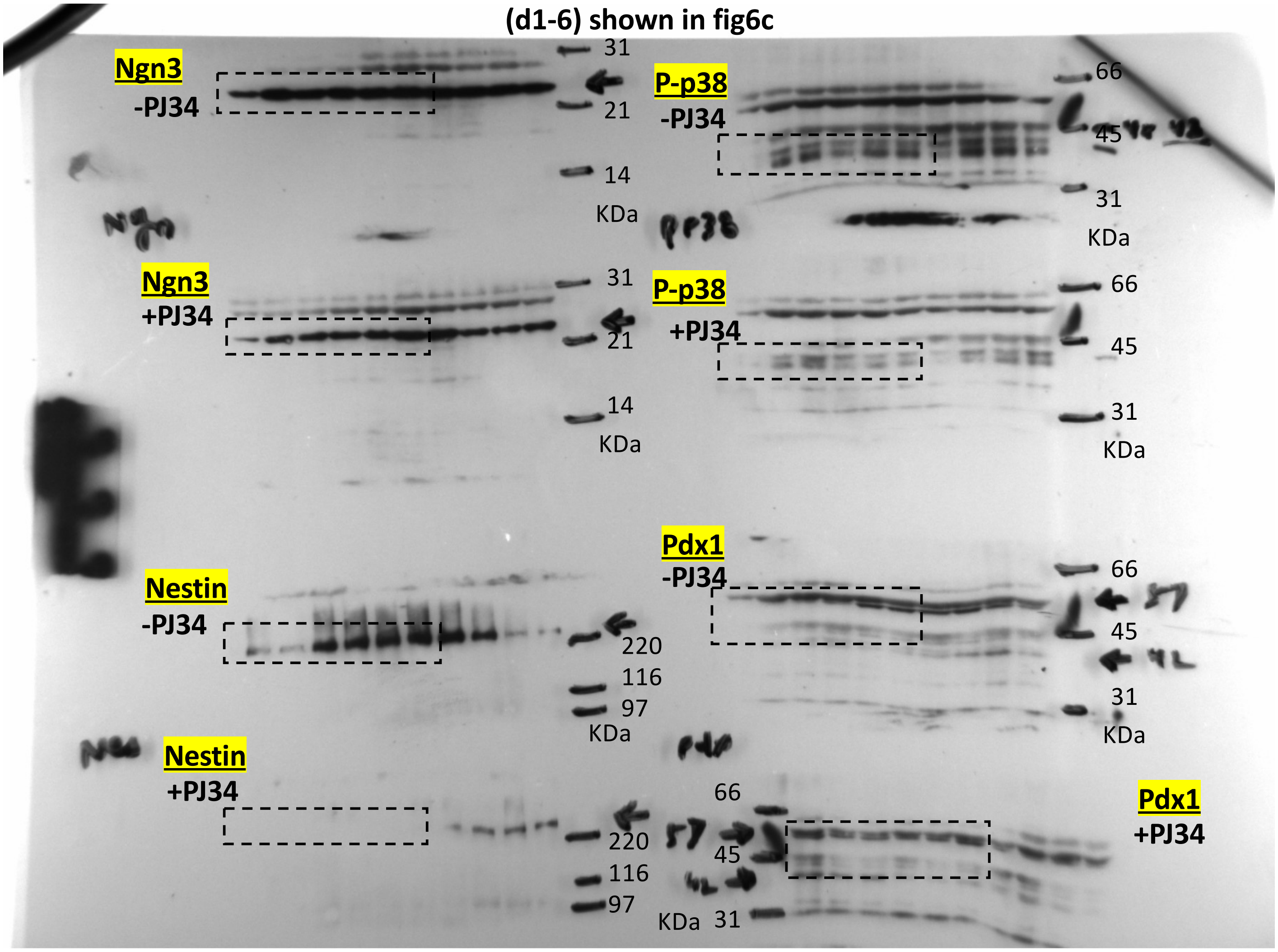

Supplementary Figure 17. Uncropped full-length immunoblot images from 10-day time course study to show original source data for Ngn3, P-p38, Nestin and Pdx1 proteins in PANC-1 differentiated clusters with SFM control and activin-A in presence or absence of PJ34 as shown in fig 6c.

Uncropped ponceau blot images of differentiation markers in time course study (d1-6) shown in fig6c

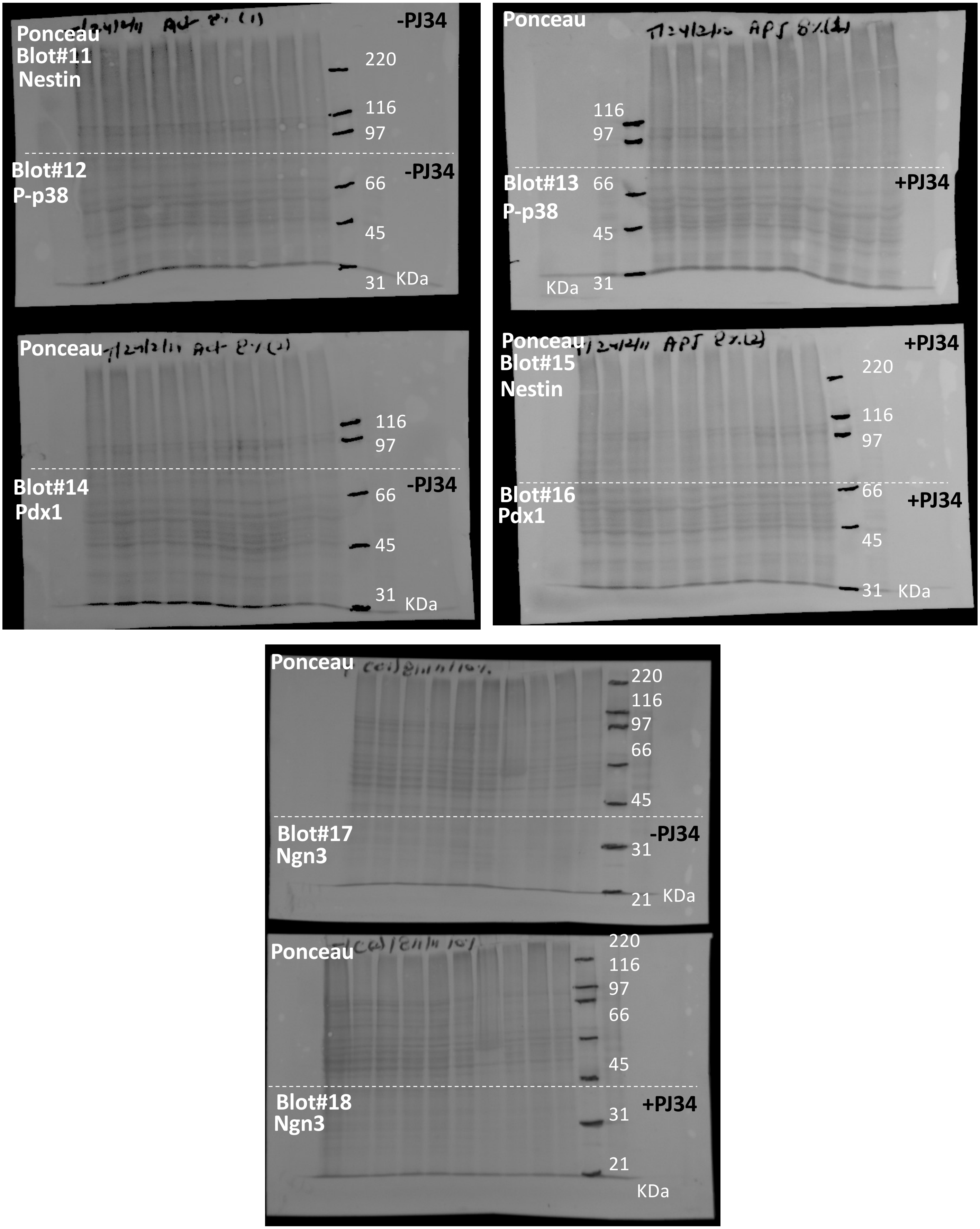

Supplementary Figure 18. Corresponding uncropped ponceau stained blots to show the original source data for Nestin, Ngn3, P-p38 and Pdx1 protein in lysates from PANC-1 differentiated clusters with SFM control and activin-A in presence or absence of PJ34 as shown in supplementary fig 17. Dashed white lines represent membrane cut at molecular weight to allow for multiple protein probing using the same blot.

Uncropped immunoblot images for differentiation markers in PANC-1 clusters from time course study (d1-6) shown in fig6c

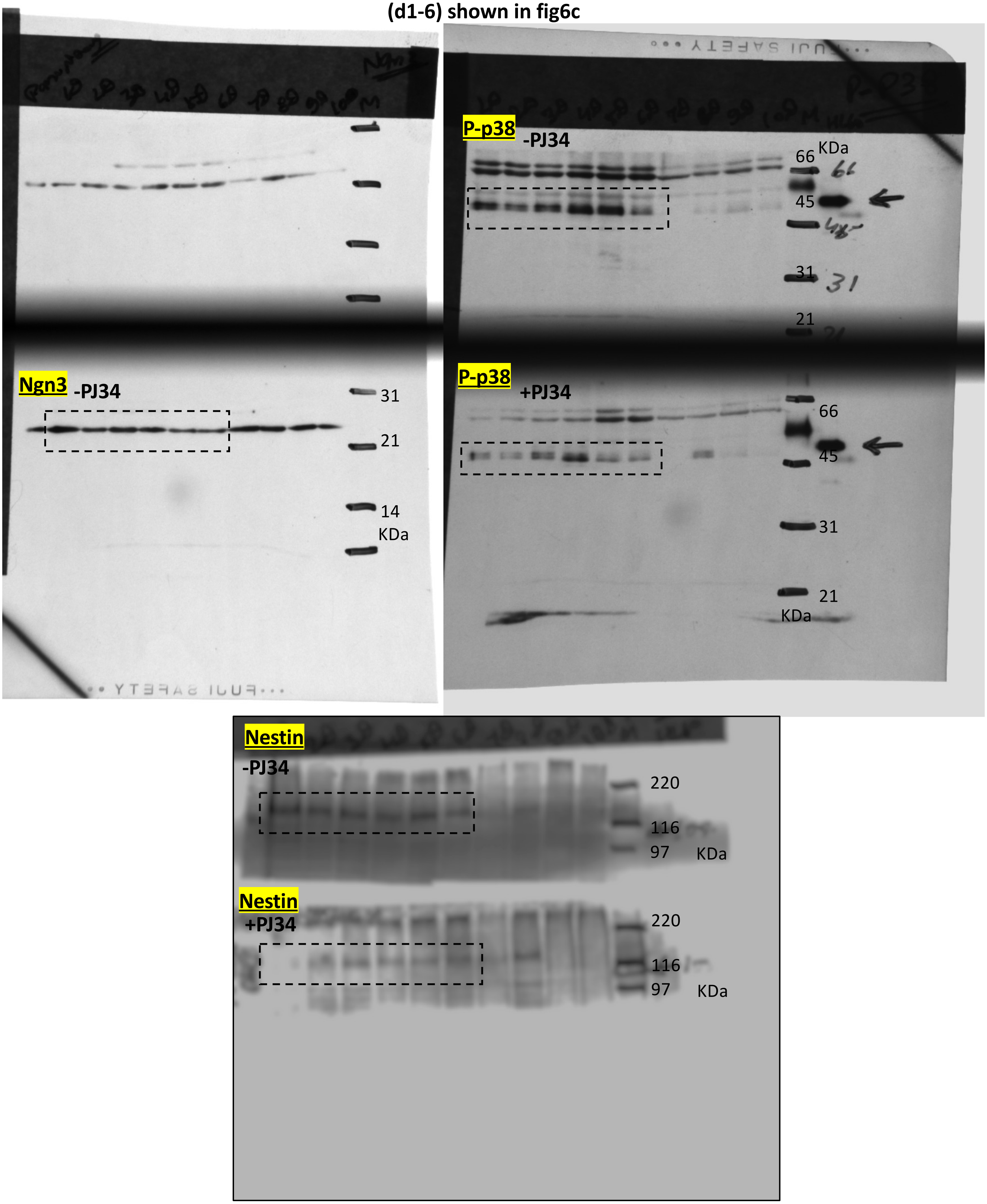

Supplementary Figure 19. Uncropped full-length immunoblot images from 10-day time course study to show original source data for Ngn3, P-p38, and Nestin proteins in PANC-1 differentiated clusters with SFM control and activin-A in presence or absence of PJ34 as shown in fig 6c.

Uncropped ponceau blot images of differentiation markers in time course study (d1-6) shown in fig6c

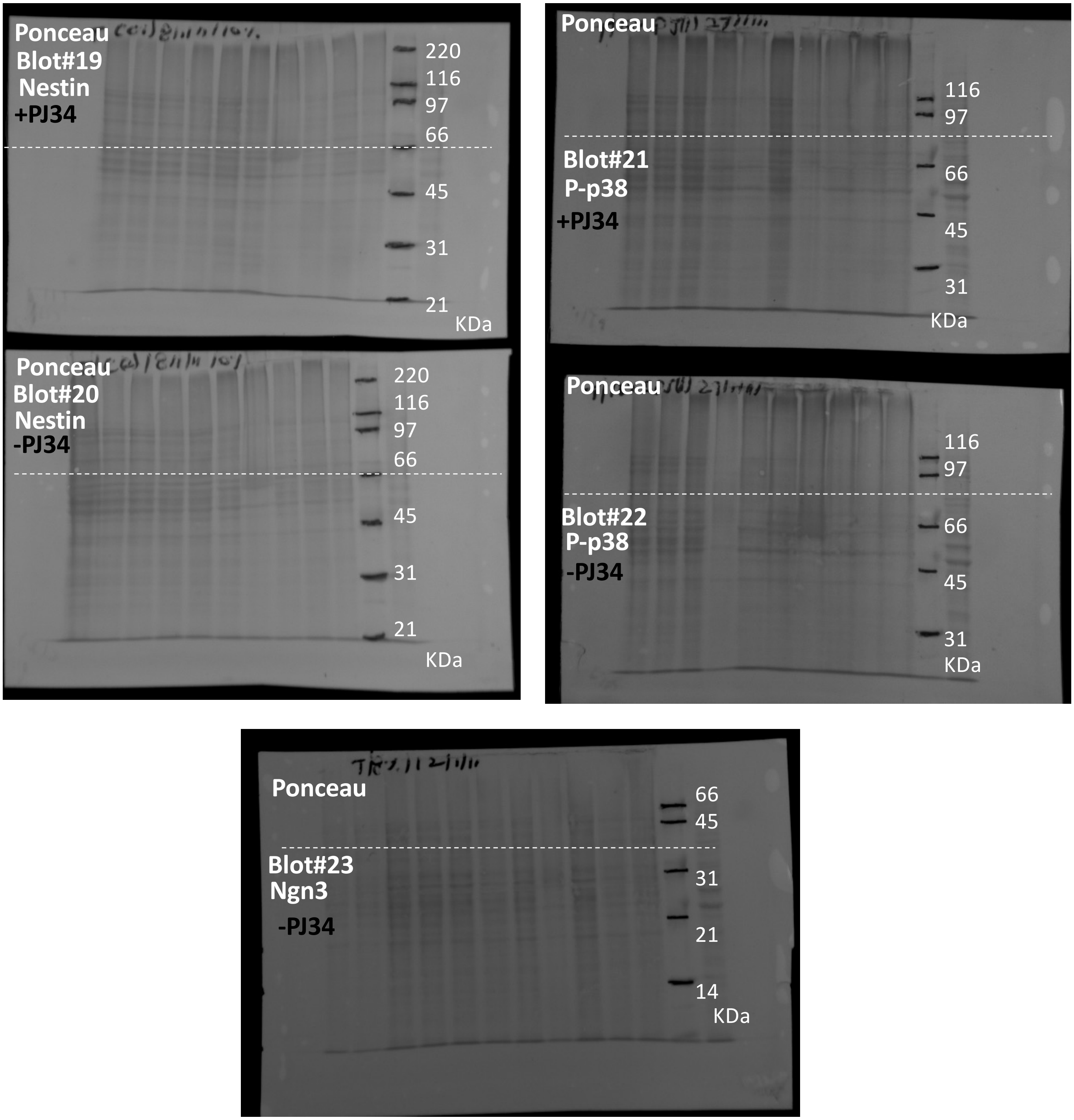

Supplementary Figure 20. Corresponding uncropped ponceau stained blots to show the original source data for Nestin, Ngn3, and P-p38 protein in lysates from PANC-1 differentiated clusters with SFM control and activin-A in presence or absence of PJ34 as shown in supplementary fig 19. Dashed white lines represent membrane cut at molecular weight to allow for multiple protein probing using the same blot.
